# Atypical memory B cells from natural malaria infection produced broadly neutralizing antibodies against *Plasmodium vivax* variants

**DOI:** 10.1371/journal.ppat.1012866

**Published:** 2025-01-23

**Authors:** Piyawan Kochayoo, Saya Moriyama, Ryutaro Kotaki, Pongsakorn Thawornpan, Chayapat Malee, Chaniya Leepiyasakulchai, Francis Babila Ntumngia, John H. Adams, Yoshimasa Takahashi, Patchanee Chootong

**Affiliations:** 1 Department of Clinical Microbiology and Applied Technology, Faculty of Medical Technology, Mahidol University, Bangkok, Thailand; 2 Research Center for Drug and Vaccine Development, National Institute of Infectious Diseases, Shinjuku, Tokyo, Japan; 3 Center for Global Health and Inter-Disciplinary Research, College of Public Health, University of South Florida, Tampa, Florida, United States of America; 4 Institute for Vaccine Research and Development, Hokkaido University, Sapporo, Hokkaido, Japan; University of Arkansas for Medical Sciences, UNITED STATES OF AMERICA

## Abstract

Expansion of atypical memory B cells (aMBCs) was demonstrated in malaria-exposed individuals. To date, the generation of *P*. *vivax*-specific aMBCs and their function in protective humoral immune responses is unknown. Here, *P*. *vivax* Duffy Binding Protein II (PvDBPII) probes were generated to detect the development and durability of specific aMBCs, and to demonstrate the capacity of these cells to produce neutralizing antibodies following natural infections. PvDBPII-specific aMBCs were elicited during malaria illness, and they persisted through the recovery phase of infections. To address biology and function of *P*. *vivax*-specific aMBCs in producing protective antibodies, a single MBC was cultured, and the secreted IgG was tested for binding and inhibition activity. The aMBC-derived clones produced antibodies with variable levels of anti-PvDBPII IgG in cultures, and some produced high antibody levels comparable to classical MBC clones. Thus, we focused our attention on the function of aMBCs in producing neutralizing antibodies. Among the aMBC clones, A1F12 and B4E11 produced broadly neutralizing antibodies against a panel of PvDBPII variants. Notably, B cell receptors (BCRs) of PvDBPII-specific aMBCs expressed unique IGHV genes, with similar usage of IGHV1-3, comparable to classical MBCs. The somatic hypermutation (SHM) rate and CDR3 length of VH and Vκ in these two MBC subsets were not significantly different. Together, our findings revealed that *P*. *vivax* infections elicited the development and persistence of *P*. *vivax*-specific aMBCs. The accumulation of aMBCs during and following infections might play an important role in producing protective antibodies against malaria.

## Author summary

The desired outcome of a malaria vaccine is to induce long-term protective immunity. Malaria-specific memory B cells (MBCs) develop during acute infections. However, the durability of these specific MBC responses and whether they are stable or short-lived after parasite clearance remain unclear. Expansion of aMBCs was reported to interfere with anti-malarial antibody responses. Here, we developed a *P*. *vivax* Duffy Binding Protein II (PvDBPII) antigen probe to detect specific MBCs in *P*. *vivax*-infected subjects and then to deeply assess the capacity of aMBCs to produce neutralizing antibodies. Natural infections with *P*. *vivax* elicited PvDBPII-specific aMBCs, and these cells persisted at least 6–9 months after infections. We firstly demonstrated at the single cell level that *P*. *vivax*-specific aMBCs produced antibodies. IgG and human monoclonal antibodies derived from these cells were highly efficient in broadly inhibiting diverse PvDBPII variants. Of note, BCR analysis of *P*. *vivax*-specific aMBCs showed uniqueness in IGHV genes while sharing common IGHV1-3 usage and similarities in SHM rates and CDR3 lengths compared to classical MBCs. These data suggest that aMBCs expressing different BCR characteristics are elicited in response to *P*. *vivax* antigen stimulation. Together, our data provide new insights into the responses of aMBCs following *P*. *vivax* infections and their production of protective antibodies. Such understanding will be important in the development of an efficacious malaria vaccine that induces broad and long-lasting immune protection.

## Introduction

*Plasmodium vivax* is the most widespread human malaria parasite. It predominates outside Africa, especially in Asia and Latin America, with more than 14 million cases occurring each year. More than one third of the global population is at risk of vivax malaria [1]. Given the rapid spread of drug-resistant parasite strains and the formation of hypnozoites in the liver with potential to cause relapse and the diversity of antigens, a highly effective vaccine to prevent the disease is essential [2,3]. Antibodies play a crucial role in controlling malaria infections [4,5]. Naturally acquired antibody responses against *P*. *vivax* infections increase with age as a result of repeated exposure to the parasite [6,7]. These antibodies do not prevent infection but do decrease parasite density, the frequency of clinical symptoms and disease severity [8]. In the blood stage of malaria, the binding of antibodies to parasite antigens disrupts the interactions between parasite ligands and cognate host receptors required for red blood cell invasion [5,9]. Among blood stage antigens, the *P*. *vivax* Duffy Binding Protein region II (DBPII) is the central molecule necessary for the invasion of reticulocytes through its ability to bind Duffy Antigen Receptor for Chemokines (DARC) on the reticulocyte surface [10]. The genetic polymorphism within this protein is known to alter its antigenic character with induction of short-lived and allele-specific naturally acquired anti-DBPII antibodies [9,11,12]. Strain-transcending humoral immunity has been detected in only a few individuals despite repeated exposures [8,13]. Based on these concerns, a successful DBPII-based vaccine strategy relies on targeting relatively conserved antigenic epitopes to induce broadly neutralizing antibodies.

A successful malaria vaccine needs to be able to elicit long-term protective immunity. The function and longevity of anti-malarial antibodies are highly variable, with some individuals acquiring long-term protection following a limited number of exposures, whereas others may require repeated exposures to generate such protective immunity [14,15]. This observation has led to the hypothesis that the slow and imperfect acquisition of humoral immunity to malaria is associated with impaired development of MBCs or antibody secreting cells (ASCs). During acute vivax malaria, MBCs are detected which are specific to the parasite’s pre-erythrocytic or blood stage antigens [14,16–19]. These *P*. *vivax*-specific MBCs persist in subjects who recover from infections, some for as long as 3–4 years [14,17], indicating a capacity of *P*. *vivax* parasites to induce the development and maintenance of MBCs. However, a critical knowledge gap remains regarding the durability of *P*. *vivax*-specific MBC responses and whether repeated infections are necessary. If so, how frequent is boosting necessary for acquisition of long-lasting and functionally active MBC responses.

In the context of short-lived MBC responses to malaria, the accumulation of aMBCs has been associated with the poor acquisition of long-term immunity to *Plasmodium* infections. Expansion of aMBCs (CD21^-^CD27^-^Tbet^+^) was found in chronic *P*. *falciparum* exposure in regions of high malaria endemicity [20–22]. These cells overexpress inhibitory receptors (FCRL5, CD11c and CXCR3), and have a reduction in the B-cell receptor (BCR) signaling pathway, leading to ineffective B cell production of antibodies [20,21]. However, a recent study showed that aMBCs responded to membrane-associated antigens [23]. Comparing the stimulation of aMBC responses by low-versus high-affinity antigens showed little or no difference, indicating that aMBCs selectively reduced responses to low-affinity antigens [24]. Additionally, aMBCs produce antibodies with the help of T follicular helper (Tfh) cells [25]. In vivax malaria, an increased frequency of aMBCs was observed during both the acute and recovery phases of infections [14,26–28]. Through study of *in vitro* cultures, aMBCs were shown to secrete immunoglobulin after receiving IFN-γ and IL-21 signals [26]. These previous reports were based on bulk aMBCs, of either CD21^-^CD27^-^ or CD21^-^CD27^-^Tbet^+^FCRL5^+^ phenotype. The specificity of these expanded aMBCs, and the function of *P*. *vivax*-specific aMBCs are unknown. Our generation of fluorochrome-labeled *P*. *vivax* antigens, along with staining for aMBC markers, should provide insights into the development of these *P*. *vivax*-specific aMBCs and their function.

In the present study, we demonstrate specificity of aMBC responses and the role of *P*. *vivax*-specific aMBCs in producing neutralizing antibodies following natural infections. The leading vaccine candidate, PvDBPII was taken to generate tetramer probes to detect antigen-specific aMBCs during the acute and recovery phases of infections, demonstrated the capability of aMBCs to produce neutralizing antibodies, and assessed the potential inhibitory activity of aMBC-derived IgG and human monoclonal antibodies (HuMoAbs) in inhibition of erythrocyte binding by a panel of PvDBPII allelic variants. Our findings will help guide the design strategy for a *P*. *vivax* malaria vaccine which will overcome the challenges posed by polymorphic antigens.

## Results

### Natural infections elicited *P*. *vivax*-specific aMBCs

**Fig 1:**
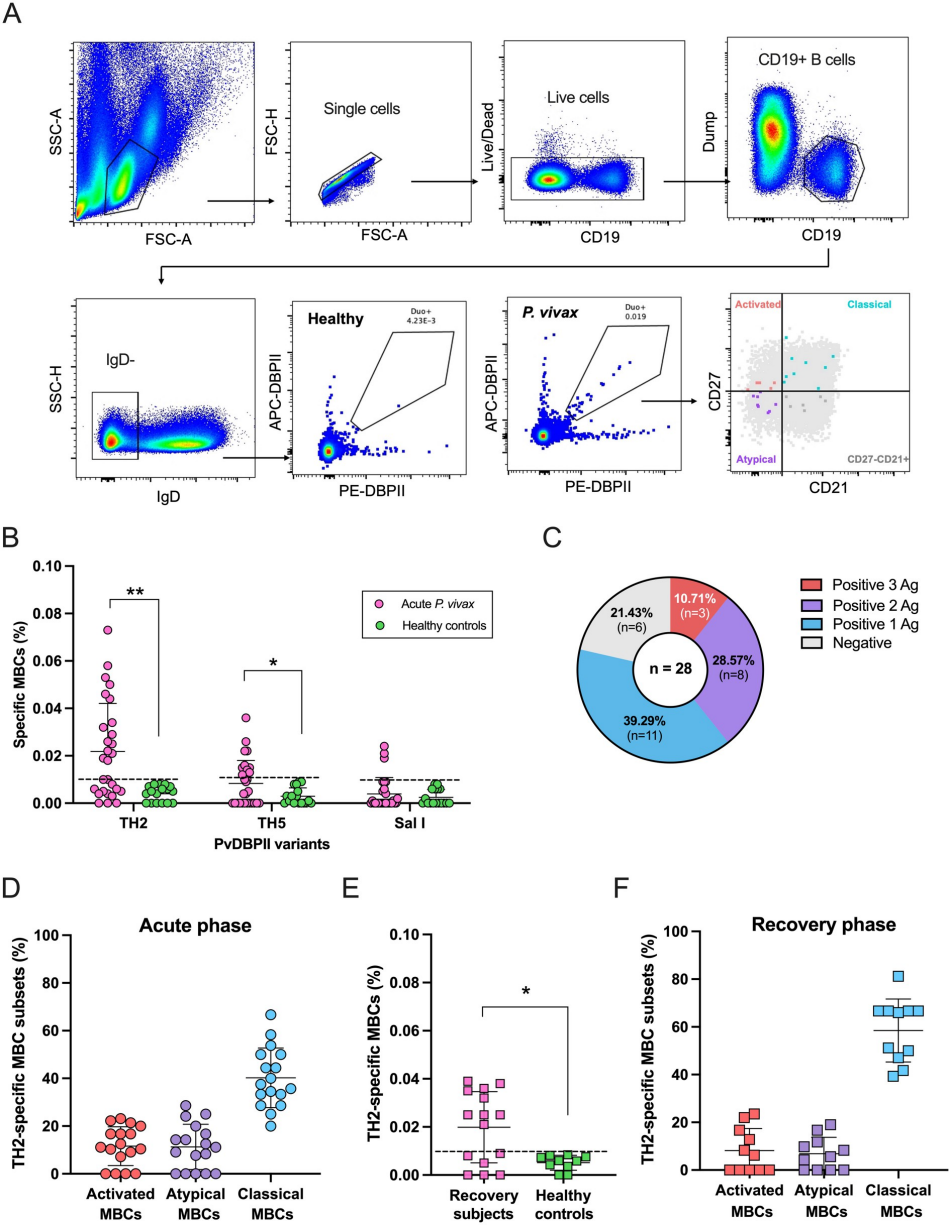
The responses of *P*. *vivax*-specific atypical MBCs (aMBCs) elicited by natural infections. **(A)** The displayed gating strategy identifies antigen-specific MBCs in *P*. *vivax* patients compared to healthy controls. CD19 and IgD were used to classify the MBC population and dual antigen tetramers to identify *P*. *vivax*-specific MBCs. MBC subsets were distinguished based on CD21 and CD27 expressions. **(B)** The frequencies of MBC specific to PvDBPII-TH2, -TH5 and reference strain Sal I from 28 acute vivax patients and 17 healthy controls are shown. **(C)** Pie graph presents the percentage of positive MBC responses to three strains of PvDBPII. **(D)** The frequencies of *P*. *vivax*-specific MBC subsets (activated, atypical and classical) detected in the patients (n = 17). **(E)** The frequency of specific MBCs in 15 subjects who after recovery from vivax infections for 6–9 months compared to those in ten healthy controls. **(F)** The frequencies of *P*. *vivax*-specific MBC subsets (activated, atypical and classical) detected in recovered subjects. Positive specific MBC responses to PvDBPII variants were defined from patients with higher frequencies of the specific MBCs than did HCs (average+2SD of HCs). Dashed line was represented cut-off value for positive responses. Statistical testing was performed by Mann-Whitney rank test; * p < 0.05, ** p < 0.01.

Expansions of bulk aMBCs have been reported during acute *P*. *vivax* malaria [14,27,28]. However, there is still limited knowledge on the specificity of the aMBCs. Given the potential of PvDBPII as blood-stage vaccine candidate [29,30], this study used the most immunogenic variant alleles (PvDBPII-TH2, -TH5 and reference Sal I strains) [14] among Thai isolates to detect *P*. *vivax-*specific aMBCs. PBMCs from *P*. *vivax* patients (n = 28) and healthy controls (HCs; n = 17) were stained with PvDBPII tetramer probes. The gating strategy of specific IgD^-^MBCs is shown in Fig 1A. During acute infections, significantly higher frequencies of MBC-specific to PvDBPII-TH2 (average 0.022%, SD 0.020%) and PvDBPII-TH5 (average 0.008%, SD 0.010%) were observed in vivax patients compared to those in HCs (PvDBPII-TH2; average 0.004%, SD 0.003%; PvDBPII-TH5; average 0.003%, SD 0.004%) (Fig 1B). Only 6 samples showed a positive specific MBC response to the reference Sal I allele (patients; average 0.004%, SD 0.007%; HCs; average 0.003%, SD 0.003%) (Fig 1B). To address the development of MBCs against diverse variant strains of PvDBPII, we analyzed whether infected individuals had positive MBC responses to 1, 2 or all 3 PvDBPII variants. Interestingly, of the 28 patients, three (10.71%) had specific MBCs to all tested antigens, eight (28.57%) to two variant antigens and most (39.29%) to a single strain (Fig 1C).

To further analyze the phenotypes of *P*. *vivax*-specific MBCs, 17 subjects (60.71%) who had positive responses against PvDBPII-TH2 allele were selected. Of their MBCs, 11.61%, 11.28%, and 40.24% exhibited activated MBC (CD21^-^CD27^+^), aMBC (CD21^-^CD27^-^), and classical MBC (CD21^+^CD27^+^) phenotypes, respectively (Fig 1D). In addition, we determined whether *P*. *vivax*-specific MBCs persisted after parasite clearance. Fifteen independent subjects who had recovered from infections for 6–9 months were recruited. The frequencies of PvDBPII-TH2-specific MBCs in these subjects (average 0.019%, SD 0.014%) was significantly greater than in HCs (n = 10; average 0.005%, SD 0.003%) (Fig 1E). Among positive PvDBPII-TH2-specific MBC responders, the activated, atypical and classical MBCs were presented in 6 (54.55%), 7 (63.64) and 11 (100.00%) subjects, respectively (Fig 1F). In addition, the presence of PvDBPII-TH2-specific aMBCs did not correlate with age (r = -0.3100, p = 0.2259), while a positive correlation with age was observed in PvDBPII-TH2-specific classical MBCs (r = 0.5093, p = 0.0368) (S1 Fig).

### Clones of aMBCs revealed heterogeneity in produced IgG antibody

**Fig 2:**
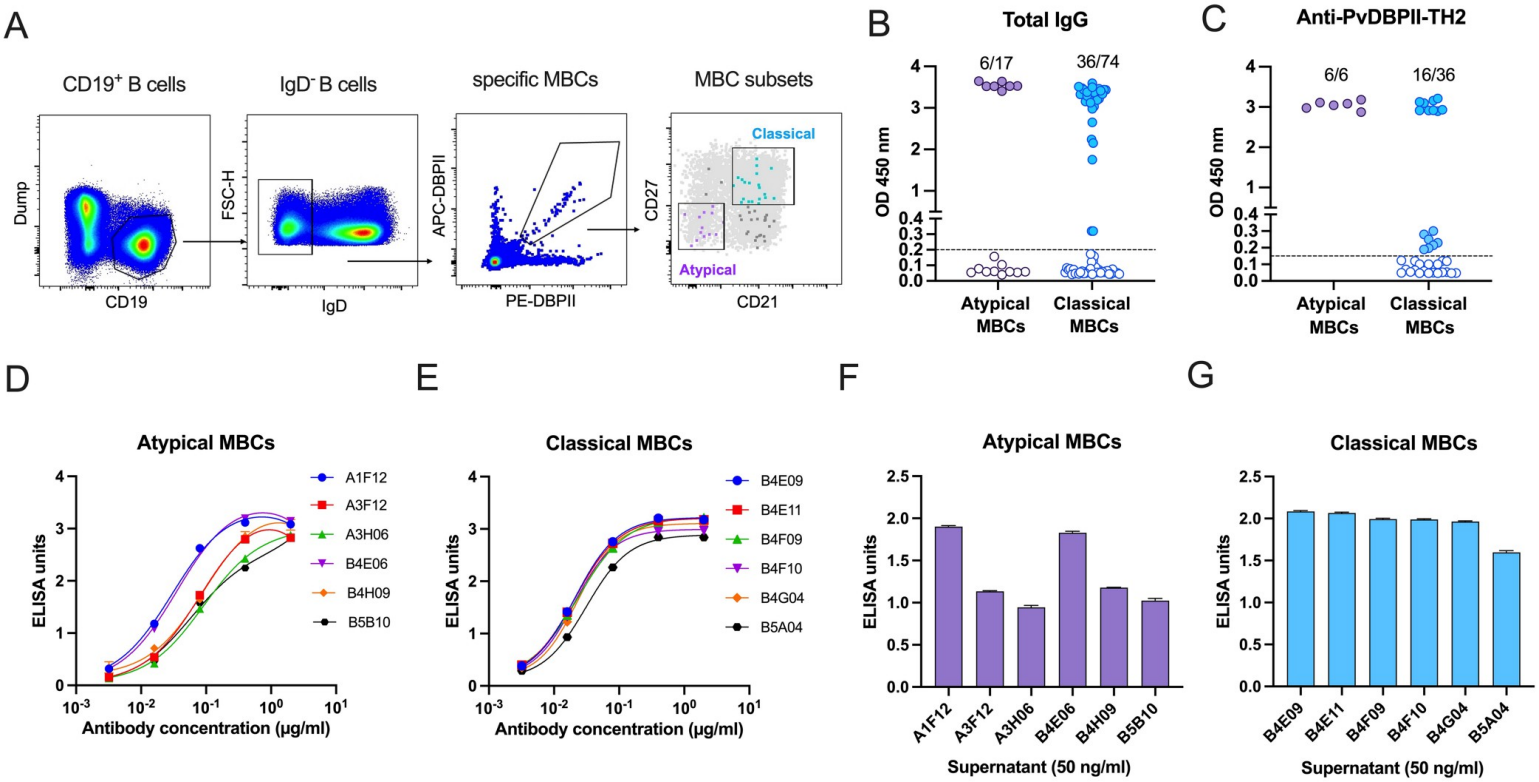
Levels of anti-*P*. *vivax* IgG antibody produced by atypical MBC (aMBC) clones. **(A)** Representative gating of specific aMBCs and classical MBCs for sorting. Within the CD19^+^ IgD^-^ MBC gate, specific aMBCs (purple) and classical MBCs (blue) were isolated after exclusion of non-B cells (Dump) and enrichment with duo antigen tetramers. **(B)** Total IgG and **(C)** anti-PvDBPII-TH2 antibody in aMBC and classical MBC culture supernatants. Binding reactivity of **(D)** aMBC and **(E)** classical MBC-derived IgG antibodies against the PvDBPII-TH2. Antibody levels of **(F)** aMBCs and **(G)** classical MBCs, presented as the IgG concentrations required to achieve 50 ng/ml. Bars represent average + SD. The experiment was done in duplicate wells and repeated twice.

The differentiation of aMBCs into ASCs after receiving T cell signals was reported from previous studies [25,27]. Here, we evaluated the capacity of *P*. *vivax* antigen-specific aMBCs to ASC differentiation and antibody production, compared to classical MBCs. Of 15 recovered subjects, three with the high frequency of aMBCs specific to PvDBPII-TH2 variant (Fig 1F) were selected for functional analysis. Using the duo antigen tetramer technique, PvDBPII-TH2-specific MBCs were identified (Fig 2A). Seventeen and 74 PvDBPII-TH2-specific aMBCs and classical MBCs, respectively, were sorted for *in vitro* single cell culturing [31,32]. After 24 days, the monoclonal IgG in MBC culture supernatants were screened for binding activities. The MBC clones that produced high IgG levels (OD values > average + 2SD of non-B cell wells) were defined as positive IgG^+^ MBC clones. Six aMBC and 36 classical IgG^+^ MBC clones were obtained from the cultures (Fig 2B), and carried out for detecting anti-PvDBPII-TH2 antibodies in supernatants. Six and 16 clones of aMBCs and classical MBCs, respectively, secreted antibodies against PvDBPII-TH2 antigen (Fig 2C). In addition, levels of IgG subclass in culture supernatant were detected. Almost all PvDBPII-specific clones of aMBCs and classical MBCs predominantly secreted IgG1, with only one clone of classical MBCs showing positive IgG3 (S2 Fig).

To characterize the binding activity of IgG from aMBC and classical MBC cultures, aMBC (n = 6) or classical MBC (n = 6) clones that produced high anti-PvDBPII-TH2 IgG levels were selected. The secreted IgG from each clone was determined by end-point dilution (Fig 2D and 2E). Then, a fixed IgG concentration (50 ng/ml) was used as the basis for determination of the difference in anti-PvDBPII-TH2 antibody levels (Fig 2F and 2G). We found that the aMBC clones produced a range of IgG levels (Fig 2F). Two aMBC clones produced antibodies capable of high binding to PvDBPII-TH2, as did all six classical MBC clones (Fig 2F and 2G). However, four clones of aMBCs produced anti-PvDBPII antibodies in low reactivity (Fig 2F).

### Broadly-reactive binding of aMBC-derived IgG

**Fig 3:**
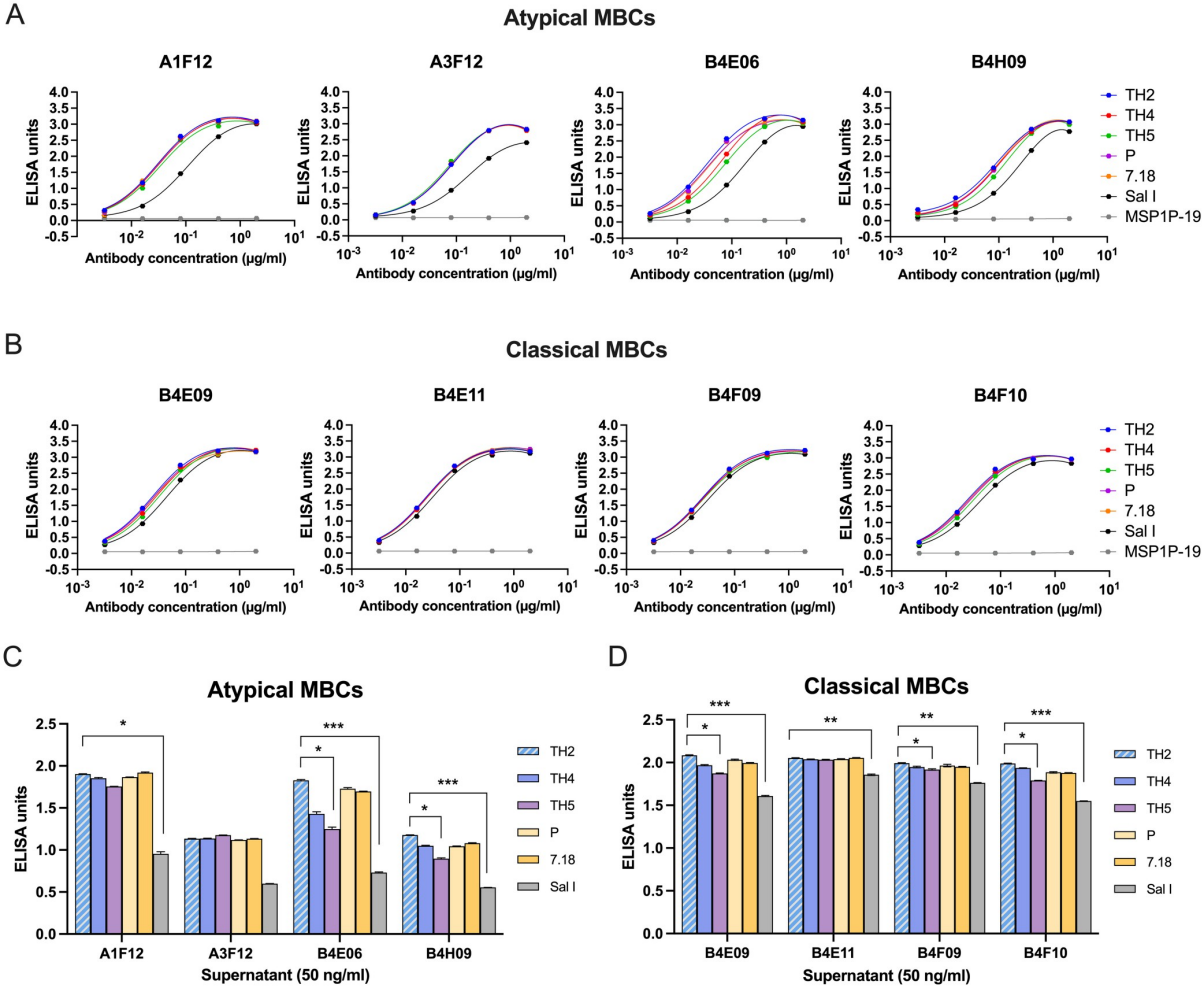
Broad binding of atypical MBC (aMBC)-derived IgG antibodies against PvDBPII variants. Reactivities of **(A)** aMBC and **(B)** classical MBC-derived IgG antibodies against a panel of variant rPvDBPII antigens are shown. End-point dilutions of each clone was determined using culture supernatants in an ELISA assessing cross-reactivity to PvDBPII variants. OD values were converted to ELISA units (EU) by normalizing with the OD of a standard [33,34]. The binding activity of IgG from **(C)** aMBC and **(D)** classical MBC clones was compared against a panel of PvDBPII variants (TH2, -TH4, -TH5, -P, -7.18) and reference SaI I strain by ELISA. Antibody level of each clone was presented as the IgG concentrations required to achieve 50 ng/ml. Antibodies that bound to all tested heterologous PvDBPII (TH4, -TH5, -P, -7.18) and homologous PvDBPII-TH2 strains were defined as broadly reactive MBC-derived IgG. Statistical testing was performed by one-way ANOVA analysis and multiple-comparison analysis by Dunn’s multiple comparisons; * p < 0.05, ** p < 0.01, *** p < 0.001. Bars represent average + SD. The experiment was done in duplicate wells and repeated twice.

Since a key challenge in development of an efficacious malaria vaccine is the antigenic polymorphism of the parasites, we assessed the generation of cross-reactive aMBCs from *P*. *vivax* infections and their competency to secrete antibodies against variant antigens. First, we characterized the binding of MBC-secreted IgG against a panel of PvDBPII strains: Thai variants (PvDBPII-TH4 and PvDBPII-TH5), PNG variants (PvDBPII-P, PvDBPII-7.18) and a reference strain (PvDBPII-Sal I). Culture supernatants with high binding reactivity of anti-PvDBPII-TH2 IgG (Fig 2F and 2G) were selected for determination of end-point dilution. Thus, both aMBC (A1F12, A3F12, B4E06 and B4H09) and classical MBC (B4E09, B4E11, B4F09, B4F10) clones were analyzed. Of the four aMBC clones, all produced high levels of antibodies against heterologous PvDBPII alleles, although there was some variability observed with B4E06 and slight variation with B4H09. Of note, they showed less reactivity with Sal I (Fig 3A). Of the four classical MBC clones, all exhibited IgG antibodies with high reactivity to all PvDBPII variants and to Sal I (Fig 3B). As expected, no secreted IgG antibodies from either aMBC or classical MBC clones bound with PvMSP1P-19 (Fig 3A and 3B).

We further compared the reactivities of secreted IgG against heterologous PvDBPII variants. Supernatants with a fixed IgG concentration (50 ng/ml) were used as the basis for determination of the differences in PvDBPII-specific antibody binding to the panel of variants. Broad reactivity was defined as the binding of IgG to all tested PvDBPII variants. The A1F12 antibodies from aMBCs exhibited high reactivity of cross-binding to all tested PvDBPII variants, which was significantly different from that of the reference Sal I strain (Fig 3C). Interestingly, A3F12 antibodies showed low antibody reactivity, but exhibited broad binding to all PvDBPII variants, while B4E06 and B4H09 exhibited variant-specific responses (Fig 3C). For classical MBCs, all antibody clones produced high reactivity of cross-binding antibodies to the different DBPII allelic variants, with exception of the Sal I variant. The B4E11 antibodies showed a broad cross-reactivity against all PvDBPII variants, with exception of the reference Sal I strain (Fig 3D). Antibodies from clones B4E09, B4F09 and B4F10 all showed variant-specific reactivities with the different PvDBPII variants. Thus, three clones (A1F12, A3F12 and B4E11) produced broadly reactive IgG antibodies. The antibodies of these clones were assessed for neutralization activity against PvDBPII-erythrocyte binding and compared with the neutralization activity of the antibodies from clones (B4E06, B4H09, B4E09, B4F09, and B4F10).

### Inhibitory aMBC clones exhibited differing levels of binding affinity to the PvDBPII-TH2 variants

**Fig 4:**
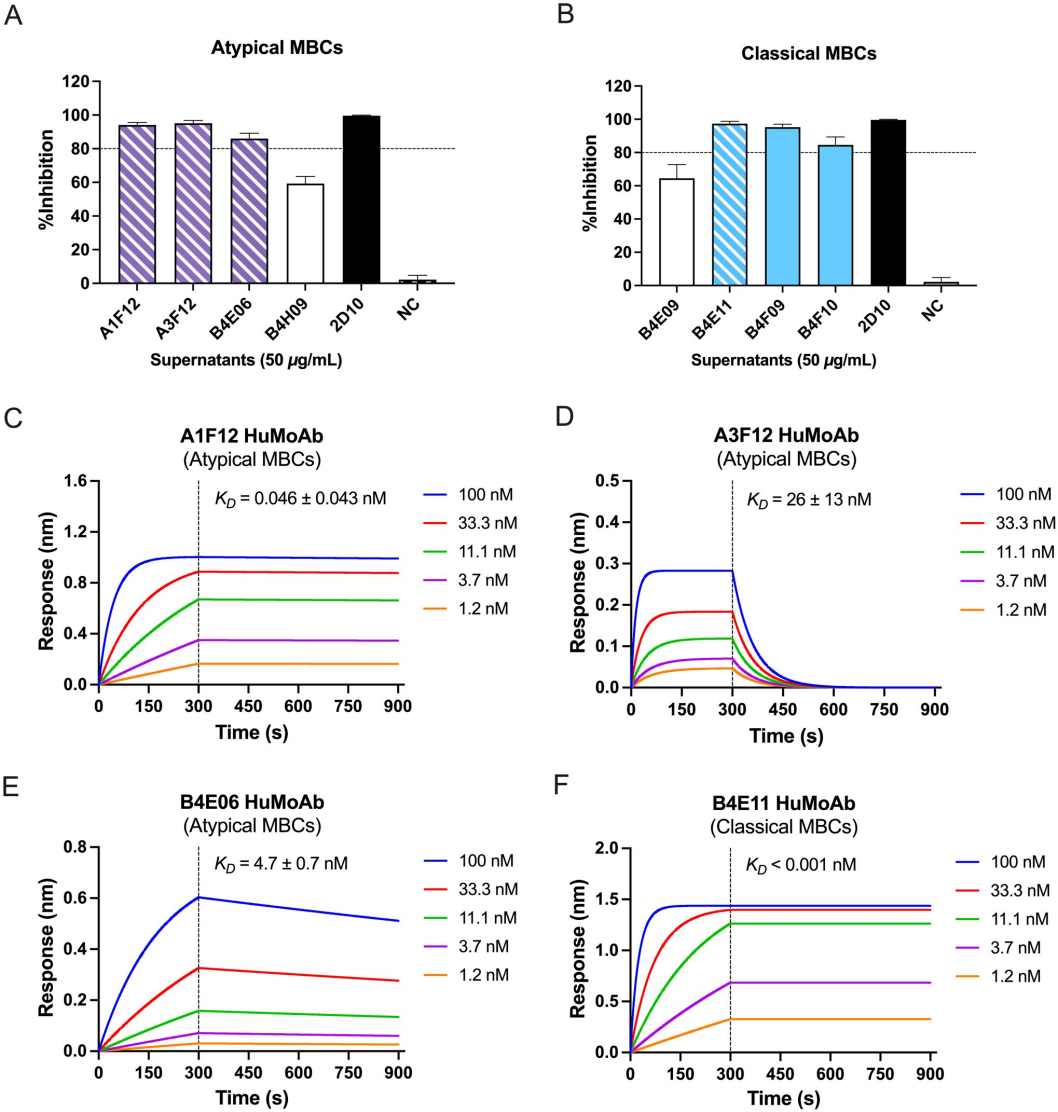
The inhibition of PvDBPII binding to erythrocytes by atypical MBC-derived IgG and binding affinity of recombinant HuMoAbs. The percentage of inhibitory IgG antibodies secreted from (**A**) aMBC and (**B**) classical MBC clones which inhibited PvDBPII-TH2 antigen binding. Bars represent average + SD. Dashed line was represented 80% cut-off value for high inhibition. The hatched bar represents percentage of inhibition by MBC-derived IgG that their MBC clones were used for HuMoAb production. (**C-F**) Binding affinity of anti-PvDBPII HuMoAbs. Fab proteins derived from HuMoAbs A1F12, A3F12, B4E06, and B4E11 specific to PvDBPII-TH2 were tested for their binding affinity against the homologous PvDBPII-TH2 antigen at different concentrations by Biolayer interferometry (BLI). Sensorgrams show association (300 s) and dissociation (600 s) of the Fab proteins.

To evaluate the ability of aMBC-secreted IgG antibodies to block PvDBPII- erythrocytes binding, we directly compared inhibition activities against the homologous PvDBPII-TH2 variant of secreted IgG at a fixed concentration (50 μg/ml). The results showed that secreted IgG antibodies from both aMBCs (A1F12, A3F12, B4E06) and classical (B4E11, B4F09, B4F10) MBCs strongly inhibited (≥ 80%) PvDBPII-erythrocyte binding while antibodies from the B4H09 aMBC and B4E09 classical MBC clones showed less inhibition (Fig 4A and 4B). As expected, 2D10 mouse monoclonal anti-PvDBPII antibody [34] had 100% of inhibition against the PvDBPII-TH2 variant (Fig 4A and 4B).

To further compare the broad neutralizing activity of the IgG produced from aMBCs and classical MBCs, we generated recombinant HuMoAbs for analysis. MBC clones were selected based on their IgG having: i) broad binding reactivity against all PvDBPII variants, and ii) high inhibitory activity against the homologous PvDBPII-TH2 strain. Thus, two aMBC clones (A1F12 and A3F12) and one classical MBC clone (B4E11) represented clones producing broadly reactive antibodies and one aMBC clone (B4E06) producing IgG with PvDBPII variant specificity were selected for HuMoAb production. We initially determined binding affinity of HuMoAbs against PvDBPII-TH2 variant antigen, using Biolayer interferometry (BLI) before testing their broad neutralizing activity. Of total four HuMoAbs, one classical MBC clone (B4E11 HuMoAb) exhibited the highest binding affinity (K_D_ < 0.001 nM), while three aMBC clones showed varying levels of binding affinity to the PvDBPII-TH2 variant. One aMBC clone (A1F12 HuMoAb) showed binding with high affinity (K_D_ = 0.046 ± 0.043 nM), comparable to the classical MBC clone, whereas two other clones showed less affinities (K_D_: A3F12 = 26 ± 13 nM, B4E06 = 4.7 ± 0.7 nM) (Fig 4C–4F and S1 Table).

### AMBC-derived HuMoAbs showed broad neutralization

**Fig 5:**
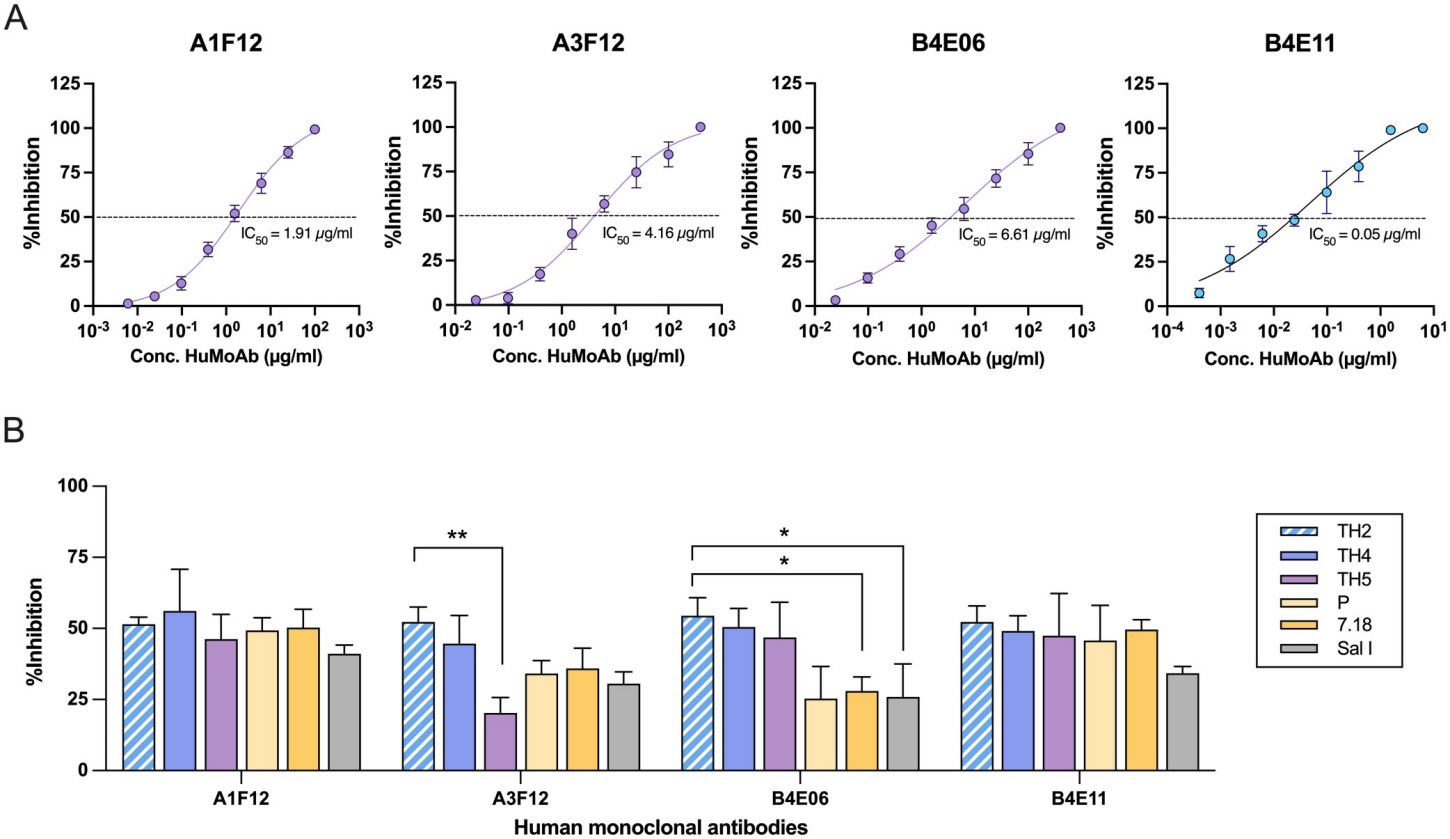
Broad inhibition of atypical MBC (aMBC)-derived HuMoAbs against binding of diverse PvDBPII variants to erythrocytes. **(A)** 50% inhibitory concentration (IC50) of anti-PvDBPII HuMoAbs. HuMoAbs A1F12, A3F12, B4E06, and B4E11 specific to PvDBPII-TH2 were tested for their inhibitory activity against the homologous PvDBPII-TH2 antigen at different concentrations by EBIA COS7 cells. Dashed line indicated 50% inhibition. Bars represent average + SD. **(B)** Transfected COS7 cells expressing PvDBPII-TH2, -TH4, TH5, -P, -7.18 or reference Sal I strains were pre-incubated with HuMoAbs at concentrations set to the IC50 of the homologous PvDBPII-TH2 variant for inhibition of PvDBPII-erythrocyte binding. The HuMoAbs that inhibited binding of all tested heterologous (TH4, -TH5, -P, -7.18) and homologous PvDBPII-TH2 strains were defined as broadly neutralizing antibodies. Statistical testing was performed by one-way ANOVA analysis and multiple-comparison analysis by Dunn’s multiple comparisons. * p < 0.05, ** p < 0.01. Bars represent average + SD. The experiment was done in duplicate wells and repeated twice.

To demonstrate the broad neutralizing activity of aMBC-derived HuMoAbs, we first confirmed inhibition activities against the homologous PvDBPII-TH2 variant of four HuMoAbs at a fixed concentration (50 μg/ml). The B4E11 antibodies had the highest inhibition (100%), while A1F12 and A3F12 and B4E06 HuMoAbs had 92.8, 82.6 and 79.5% of inhibition, respectively against PvDBPII-TH2 variant (S3 Fig). Next, the 50% inhibitory concentration (IC50) values of each HuMoAb were determined and found to be 0.05, 1.91, 4.16 and 6.61 μg/mL for B4E11, A1F12, A3F12 and B4E06 antibodies, respectively (Fig 5A). To characterize the breadth of neutralization of these HuMoAbs, their ability to inhibit a panel of PvDBPII variants from binding to human erythrocytes was evaluated. The A1F12 and B4E11 antibodies showed no significant differences in inhibitory activity against the variant strains (Fig 5B), indicating that the conserved epitopes shared among tested PvDBPII variants were target of these neutralizing antibodies, while A3F12 and B4E06 antibodies were variant-specific inhibitory activity. The inhibition activity of A3F12 antibodies were significantly different against homologous PvDBPII-TH2 and heterologous TH5 variants, while activity of B4E06 antibodies was significantly different between the homologous PvDBPII-TH2 variant, and heterologous PNG 7.18 as well as the reference Sal I variants (Fig 5B).

### *P*. *vivax*-specific aMBCs expressed unique BCR characteristics and shared IGHV1-3 usage with classical MBCs

**Fig 6:**
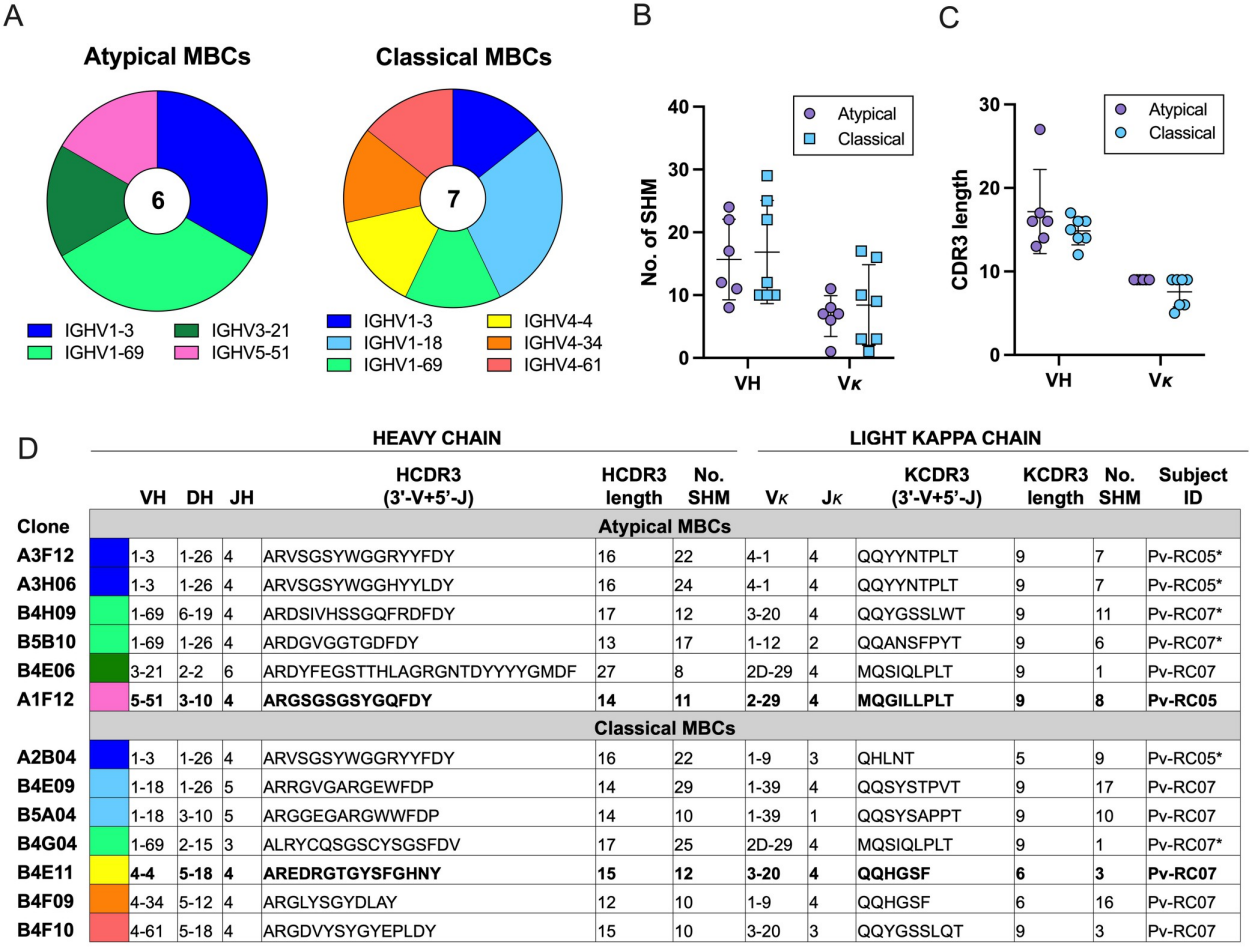
BCR repertoires of *P*. *vivax*-specific atypical MBCs (aMBCs) and classical MBCs. (**A**) The proportion of different clonal groups of 6 PvDBPII-specific aMBC and 7 classical MBC clones which were sequenced for IGHV genes from one individual are shown (color coded). (**B**) Somatic hypermutation (SHM) number and (**C**) CDR3 length of variable region genes of the heavy chain (VH) and light kappa chain (Vκ) of the specific aMBC and classical MBC clones are shown. (**D**) V(D)J usage and CDR3 sequences of PvDBPII-specific aMBC and classical MBC clones. Asterisk (*) indicates the subjects who have similar usage of IGHV (IGHV1-3 or IGHV1-69). Sequences in bold font are from two broadly reactive PvDBPII-specific antibodies (A1F12 and B4E11). Statistical testing was performed by Mann-Whitney rank test.

To investigate the diversity of *P*. *vivax*-specific aMBCs, we amplified and sequenced heavy- and light-chain V(D)J rearrangements of PvDBPII-TH2-specific aMBCs (n = 6) and classical MBC clones (n = 7) with high binding activity from two subjects (Pv-RC05 and Pv-RC07). This revealed four and six distinct clonal groups of aMBC and classical MBC clones, respectively. Two clonotypes (IGHV1-3 and IGHV1-69) were shared in these two MBC subsets (Fig 6A). In addition, our analysis showed that aMBCs and classical MBCs had similar somatic hypermutation (SHM) numbers and CDR3 lengths of variable heavy (VH) and variable light kappa (Vκ) sequences with no statistically significant differences (p > 0.05). The mean of the SHM numbers was 15.67 for VH and 6.67 for Vκ of aMBCs compared with 16.86 for VH and 8.43 for Vκ of classical MBCs (Fig 6B). The mean CDR3 lengths of aMBCs and classical MBCs were similar for the VH (17.17 versus 14.86) and Vκ (9.00 versus 7.57) (Fig 6C).

To understand the clonal lineage of *P*. *vivax*-specific aMBCs and classical MBCs, we further analyzed the CDR3 sequence in IGHV1-3 and IGHV1-69 which were shared. Greater than 87.5% similarity was found in IGHV1-3 clonotype of the aMBC and classical MBC subsets in Pv-RC05 subject (Fig 6D and S2 Table), suggesting that natural *P*. *vivax* infections promote the common usage of IGHV shared between CD27^-^ and CD27^+^ MBCs. However, similarity was less than 38.5% in the IGHV1-69 group in Pv-RC07 subject, making different progenitors more likely (Fig 6D and S2 Table). In addition, two broadly inhibitory PvDBPII-specific HuMoAbs were compared for their VDJ sequences, SHM rates and CDR3 length. Clone A1F12 antibodies utilized IGHV5-51 while B4E11 used IGHV4-4 gene segments, indicating these antibodies were not derived from a common progenitor (Fig 6D). However, SHM rate and CDR-H3 length of A1F12 HuMoAbs (SHM rate = 11, CDR-H3 length = 14) did not differ from those of B4E11 (SHM rate = 12, CDR-H3 length = 15) (Fig 6D).

## Discussion

Expansions of bulk aMBC phenotypes have been demonstrated in malaria-exposed individuals in high *P*. *falciparum* malaria transmission settings of Africa [20–22], as well as in low *P*. *vivax* transmission regions of Brazil and Thailand [14,26–28]. It is unclear whether these expansions of aMBCs and their activation upon receiving T cell help could contribute to development of protective immunity against malaria. Identifying MBC subsets or phenotypes that play a major role in producing protective antibodies will facilitate development of effective and long-lasting malaria vaccines. Here, we demonstrated the generation of antigen-specific aMBCs in vivax malaria. In acute infections, PvDBPII-TH2-specific MBCs were detected in 17 individuals (average 0.024%, SD 0.020%). Of these, 11.28% and 40.24% expressed aMBC and classical MBC phenotypes, respectively. Thus, we focused our study on the durability of aMBC responses. These were observed during the recovery phase in 7 subjects indicating that *P*. *vivax* infections elicited the development and persistence of antigen-specific aMBCs. The finding was consistent with similar observations in *P*. *falciparum* malaria [25,35,36]. The PfAMA1- and PfMSP1-specific aMBCs were detected in chronic *P*. *falciparum*-exposed individuals in Mali and Uganda [25,35]. To determine the factors that may drive the development of aMBCs, we assessed whether the presence of *P*. *vivax*-specific aMBCs increased with age and number of infections. No correlation was observed between the frequency of specific aMBCs and age (r = -0.3100, p = 0.2259). Additionally, almost all of our *P*. *vivax* participants were diagnosed with the first *P*. *vivax* infection. There was only one acutely infected patient and one recovered subject who had repeated episodes of *P*. *vivax* malaria. The time intervals to reinfection or relapse were 12 and 3 months, respectively (S3 Table). Of note, the intensity of malaria transmission might impact the accumulation of malaria-specific aMBCs. The number of bulk aMBCs (not antigen-specific) was high in Malian adults and children with chronic asymptomatic *P*. *falciparum* infections, compared to uninfected children, suggesting that the long-term exposure to parasite may drive the expansion of aMBCs [21,22]. Here, in a low malaria transmission setting, we found that *P*. *vivax*-infected individuals with the first episode of clinical symptoms developed antigen-specific aMBCs. Thus, an acquisition of aMBC responses to malaria was not associated with repeated or chronic exposure to parasites. Our evidence was supported by a previous observation reporting that an expansion of atypical MBCs was a normal part of the humoral immune response to both malaria and influenza vaccinations [37]. Therefore, further investigation into the factors influencing the development and activation of malaria-specific aMBCs and the correlation of aMBC frequency with malaria protection or reduced severity would be valuable for improving malaria vaccines.

Given the highly polymorphic nature of human *Plasmodium* parasites, the development of cross-reactive MBC responses against variant antigens is an important key for vaccine strategies. Here, we highlight the generation of MBCs against diverse variant strains of *P*. *vivax* antigen following natural infection. Of the 28 subjects with acute malaria, three produced MBC responses to all tested antigens (PvDBPII-TH2, -TH5, and reference Sal I). Most patients produced strain-specific MBC responses; 25.00%, 10.71% and 3.57% specific to PvDBPII-TH2, -TH5, and Sal I strains, respectively. Our data suggested that natural *P*. *vivax* infection elicited MBC responses against various endemic *P*. *vivax* variant strains. To support the capability of MBCs to produce cross-reactive antibodies, single clones of PvDBPII-TH2-MBCs were cultured and then tested for broad binding and inhibition of a panel against PvDBPII allelic variants. Screening of IgG culture supernatants showed that two (A1F12 and A3F12) clones and one (B4E11) clone from aMBCs and classical MBCs, respectively, showed cross-reactivity. Most significantly, MBC-derived HuMoAbs from clones A1F12 and B4E11 showed broadly inhibitory activity against a panel of PvDBPII variants. These data support the previous finding that strain-transcending anti-PvDBPII antibodies were acquired in natural infections [38–40]. Overall, our study highlights the development of a small number of broadly reactive aMBCs and classical MBCs against *P*. *vivax* variant strains following natural infections. The differentiation of these MBC subsets into ASCs potentially leads to potential production of broadly neutralizing antibodies against *P*. *vivax* parasite binding and invasion of human erythrocytes.

Although expansion of aMBCs in malaria has been reported [14,20,27,41], the competency of these cells to differentiate into ASCs for secreting protective anti-malarial antibodies remains unknown. Here, we used single MBC cultures (Nojima cultures) to demonstrate *P*. *vivax*-specific aMBC function in ASC differentiation. The culture system contains MS40L-low feeder cells and multiple cytokines (IL-2, IL-4, IL-21 and BAFF) [31,32]. Our result demonstrated that *P*. *vivax*-specific aMBCs differentiated into ASCs for IgG secretion. However, in comparison to classical MBCs, they had lower ability in ASC differentiation. The IgG reactivity in MBC culture supernatant showed that 35.29% (6/17) and 48.64% (36/74) of aMBC and classical MBC clones were positive, respectively (Fig 2B). Of note, the phenotypes of *P*. *vivax*-specific aMBCs were heterogeneous (S4 Fig), consistent with a previous study in chronic *P*. *falciparum* parasite exposure [35], in which three subsets of aMBCs were identified, but not all developed into ASCs. Of total six PvDBPII-TH2-specific aMBC clones, three clones (A1F12, A3F12 and B4E06) differentiated and secreted IgG with high inhibitory efficacy. Notably, one clone (A1F12) exhibited high binding affinity, comparable to that of the classical MBC clone (B4E11), and produced broadly inhibitory antibodies against the binding of diverse PvDBPII variants to human erythrocytes. Together, our findings highlight a protective role of aMBCs in humoral immune response to malaria by differentiating into ASCs for producing broadly neutralizing antibodies against *P*. *vivax* invasion into human erythrocytes. However, low competency of aMBCs in ASC differentiation was detected, compared to classical MBCs. This finding is consistent with previous *in vitro* studies that the differentiation of aMBCs into ASCs required signals (IL-21 cytokine) and interactions with Tfh cells [25,27]. In addition to their role as precursors of ASCs, as shown in previous studies and ours, aMBCs have capacity to present antigens by internalizing membrane-associated antigens [23] and express MHC class II and CD11c molecules to interact with T cells [42]. Also, they primed Tfh cells in Germinal Center (GC) to sustain the immune response in persistent malaria infection [43]. Therefore, it seems that aMBCs act as a key player in immune responses (both innate and adaptive immunity) against malaria. Investigation of mechanisms and dependencies driving malaria-specific MBCs in antigen presentation, cytokine secretion and ASC differentiation will be beneficial for vaccine design.

The BCR characteristics of aMBCs in *P*. *falciparum* malaria has been described [21,44]. However, most reported BCR repertoires are based on analyses of bulk aMBCs containing non-malaria-specific cells. Here, we first compared the usage of the IGHV gene in *P*. *vivax*-specific aMBCs and classical MBCs. Four and six IGHV genes were commonly used in aMBCs and classical MBCs, respectively. Two IGHV usages (IGHV1-3 and IGHV1-69) shared similarities in the two MBC subsets. The IGHV1-3 usage had 87.5% similarity in sequences. These data indicate that *P*. *vivax* antigen could drive the usage of diverse IGHV genes in aMBCs. The common use of IGHV1-3 in the same subject (Pv-RC05) might consider a clonal sharing between aMBCs and classical MBCs in responses to PvDBPII-TH2 antigen. In addition, we also observed the SHM rates and CDR3 lengths of VH and Vκ sequences in the *P*. *vivax*-specific aMBCs and classical MBCs. There was no significant difference in rates of SHMs and CDR-H3 lengths between aMBCs and classical MBCs. This was similar to findings in the studies of *P*. *falciparum-*experienced adults, which showed that IgG^+^ aMBCs and classical MBCs displayed similar CDR-H3 physicochemical properties and SHM rates [44,45]. Conversely, in a *Muellenbeck et al*. [46] study, the aMBCs tended to have a greater number of SHMs as compared with classical MBCs in their VH and Vκ. Of note, the accumulation of SHM numbers in aMBCs might be influenced by the age of malaria subjects, as the findings in semi-immune adults showed higher number of SHMs in aMBCs than in children [45]. In our study, unique IGHV (IGHV 3–21 and IGHV5-51) and IGKV gene usages (IGKV2D-29 and IGKV2-29) were observed in aMBCs encoding anti-*P*. *vivax* neutralizing antibodies (B4E06 and A1F12), indicating that these aMBC clones were derived from different precursors. Overall, our findings reveal that natural *P*. *vivax* infections drive development of antigen-specific aMBCs that express diverse BCR characteristics, possibly connected to different roles in immune responses against *P*. *vivax* malaria.

This study has limitations. First, we selected PvDBPII, a leading blood-stage vaccine candidate [29,30] with highly polymorphic characters in clinical studies [47], to demonstrate the development of *P*. *vivax*-specific aMBCs. However, this data may be useful for development of PvDBPII-based vaccines against diverse *P*. *vivax* variants. We did not detect MBCs specific for other *P*. *vivax* antigens or stages due to limited PBMC sample availability from our subjects. Second, the low frequency of *P*. *vivax*-specific aMBCs limited our deep profiling of the phenotypes of these cells. Use of single-cell RNA expression might help identify those cells driving the disease phenotype even when their number is small [48,49]. Third, we lacked an *in vitro* assay to test inhibitory function of human monoclonal antibody-derived aMBCs [50,51] which might confirm protective activity of clonal IgG and HuMoAbs. Fourth, we were unable to assess a possible association between the frequency of *P*. *vivax*-specific aMBCs and a subject’s number of infections, as only very few of our subjects had a history of prior infection based on records at malaria clinics. A larger cohort study would be useful to demonstrate an association of *P*. *vivax*-specific aMBCs, antibody breadth and clinical malaria protection. Future studies comparing the effects of single and multiple *P*. *vivax* parasite exposures on specific aMBC responses should be useful. Fifth, *P*. *vivax* antigen-specific IgD^-^ atypical and classical MBCs were detected in *P*. *vivax* subjects. The finding of predominant IgG1 and IgG3 subclasses in Nojima cultures did not address the ability of these two MBC subsets to undergo IgG subclass switching following natural malaria infection. Further analysis of IgG1^+^ or IgG3^+^ *P*. *vivax*-specific aMBCs or classical MBCs in *P*. *vivax* subjects is needed. Lastly, larger sample size for aMBC characterization and its ability in ASC differentiation for neutralizing antibody production, as well as V(D)J gene analysis, would better capture the variability of V(D)J gene rearrangements and their functional roles in the context of aMBC responses to *P*. *vivax* malaria.

## Materials and Methods

### Ethics statement

This study was approved by Mahidol University Central Institutional Review Board (MU-CIRB 2021/281.2505) and by the Institutional Review Board of the National Institute of Infectious Diseases (#1505). Written informed consent was obtained from each participant before blood collection. All experiments involving human subjects were conducted in accordance with relevant guidelines and regulations.

### Study design

Heparinized blood samples were taken from subjects in malaria low-transmission areas in the southern part of Thailand (Chumphon and Ranong Provinces). Twenty-eight acutely infected *P*. *vivax* patients were recruited for profiling the phenotypes of *P*. *vivax*-specific MBCs by flow cytometric analysis using PvDBPII variants (TH2, TH5, Sal I) as probes. To determine the persistence of *P*. *vivax*-specific aMBCs, 15 independent subjects previously diagnosed with *P*. *vivax* infections during an acute malaria episode and had recovered for 6–9 months (cohort study at Chumphon province) were recruited. Blood samples from these recovered subjects were collected and tested by nested PCR every three months to detect sub-patent malaria. Weekly house-to-house visits were also conducted to estimate the incidence of clinical malaria during the study period.

To demonstrate the function of *P*. *vivax*-specific aMBCs, three recovered subjects with high specific aMBC frequencies (Pv-RC03, -RC05 and -RC07) were recruited for single MBC sorting and Nojima culture. The binding and inhibitory breadth of their MBC-derived antibodies against the PvDBPII variants were determined. Six aMBC and seven classical MBC clones with high binding activity from two subjects (Pv-RC05 and Pv-RC07) were further investigated for BCR diversity analysis. Their V(D)J rearrangements were amplified and sequenced. The demographic information of recruited subjects is summarized in S3 Table.

*P*. *vivax* infections were determined by microscopic examination and confirmed with nested PCR of peripheral blood. History of prior malaria infections in subjects were obtained from records of the local malaria clinics and Vector-Borne Disease Unit 11.4. All participants were treated with a full course of primaquine and chloroquine. Twenty-seven malaria-non-infected Thai residents who lived in non-endemic areas (Bangkok, Thailand) were recruited as healthy controls (HCs).

### Production of recombinant proteins

Recombinant PvDBPII antigens from six naturally occurring alleles [TH2, TH4, TH5, P, 7.18 and Sal I (S4 Table), and rPvMSP1P-19] were expressed and purified as previously described [10]. Briefly, the genes coding for the various antigens were synthesized, codon-optimized for expression in *Escherichia coli*, and cloned into expression vector pET21a+ (Novagen) with a C-terminal 6xHis tag to facilitate purification by affinity chromatography. The resulting plasmids were transformed into *E*. *coli* BL21(DE3). Recombinant proteins were expressed, purified under denaturing conditions, and refolded to native form as previously described [10]. The refolded proteins were further purified by ion exchange chromatography, then dialyzed against PBS, aliquoted and stored at -80°C. Protein concentrations were measured by Bradford colorimetric assay using BSA as a standard. Purified proteins were validated by SDS-PAGE analysis (S5 Fig).

To produce biotinylated protein, PvDBPII DNA was cloned in an expression vector containing an N-terminal, thrombin-cleavable 6x histidine-tag, followed by a BirA site (amino acids: GLNDIFEAQKIEWHE) and a short, flexible linker sequence. Proteins were expressed as inclusion bodies, refolded and purified as described above. The purified recombinant protein was biotinylated using BirA-500 BirA biotin-protein ligase standard reaction kits (Avidity) according to manufacturing’s procedure.

### Flow cytometry with PvDBPII-specific B cell tetramer probes

To determine the frequency of cross-reactive MBCs against PvDBPII variants and their MBC subset during *P*. *vivax* infections, PBMCs from these patients and HCs were stained with antigen specific tetramers from variant DBPII alleles (PvDBPII-TH2, -TH5 and reference Sal I) and antibodies for MBC panels. Briefly, biotinylated PvDBPII variants were incubated overnight with PE-streptavidin or APC-streptavidin (Invitrogen) at 4:1.5 molar ratio at 4°C. Frozen PBMCs of patients and HCs were thawed and stained with 100 ng of each probe and 10 μM biotin for 30 min at room temperature. The cells were then stained with live/dead fixable stains and conjugated anti-human antibodies for MBC panels (anti-CD19, -CD21, -CD27, -IgD, -CD3, -CD14, and -CD16 antibodies). Non-B cells were excluded by binding to conjugated anti-human CD3, -CD14, -CD16 antibodies. All antibodies were from Biolegend (S5 Table). The analyses were done by flow cytometry (BD FACSCanto II) and data analyzed using FlowJo software version 10.10.0 (BD Biosciences). Positive specific MBC responses to PvDBPII variants were defined from patients with higher frequencies of the specific MBCs than did HCs (average+2SD of HCs).

### Single MBC sorting and culturing

Single B cells were sorted and cultured as previously described [31,32], with slight modifications. Briefly, PBMCs were stained with 100 ng of APC-labeled PvDBPII-TH2, PE-labeled PvDBPII-TH2 and 10 μM biotin for 30 min at room temperature. The cells were stained with live/dead fixable stains and conjugated anti-human antibodies (anti-CD19, -CD2, -CD4, -CD10, -CD14, -IgD, -IgG, -CD21, -CD27, -CD11c, and -FCRL5 antibodies) (S5 Table). Single CD19^+^CD2^-^CD4^-^CD10^-^CD14^-^IgD^-^IgG^+^PvDBPII-TH2^+^ B cells were sorted onto pre-cultured MS40L-low feeder cells in 96 F plates containing RPMI 1640 medium supplemented with 10% FBS, 55 μM 2-mercaptoethanol (2-ME), penicillin (100 U/mL), streptomycin (100 μg/mL), 10 mM HEPES, 1 mM sodium pyruvate, 1% minimal essential medium non-essential amino acids, recombinant human interleukin-2 (IL-2; 50 ng/mL; PeproTech), recombinant human IL-4 (10 ng/mL; PeproTech), recombinant human IL-21 (10 ng/mL; PeproTech), recombinant human B-cell activating factor belonging to the TNF family (BAFF) (10 ng/mL, PeproTech) using FACS Symphony S6 (BD Biosciences). The cultures were maintained at 37°C with 5% CO2. Half of the culture medium was replaced twice weekly with fresh medium and fresh cytokines. On day 24, culture supernatants were harvested and subjected to antibody characterization, and RNA was collected from the cells using RNeasy (Qiagen) for immunoglobulin gene sequencing. We used FACS index sorting to identify aMBC and classical MBC phenotypes of every single event sorted into a plate, prior to further functional analysis. The aMBCs and classical MBCs were classified based on median fluorescence intensity (MFI) values of CD21 and CD27 markers.

### Detection of total IgG against PvDBPII variants

Culture supernatants were evaluated for anti-PvDBPII antibody titers by indirect ELISA as previously reported [14]. Briefly, 1 μg/ml of anti-human IgG or 2 μg/ml PvDBPII variants (PvDBPII-TH2, -TH4, -TH5, -Sal I, -P, -7.18) or *Plasmodium vivax* Merozoite Surface Protein 1 paralog 19 (PvMSP1P-19) were coated on 96-well plates followed by blocking with 1%BSA-PBS. Five-fold serial dilutions of individual supernatants (starting at 2000 ng/mL of total IgG) in blocking solution were added to duplicate wells. After washing, wells were incubated with goat anti-human IgG conjugated to horseradish peroxidase (HRP) (Seracare). Signal was developed with 3,3’,5,5’-Tetramethylbenzidine (TMB) enzyme substrate (Sigma Aldrich), and optical density (OD) was read at 450 nm on a microplate reader (Thermo Scientific). Pooled plasma from *P*. *vivax* infected Thais Thai vivax plasma with high binding reactivity [14] was used as the standard calibrator on each plate. All OD values were normalized at a point on the standard curve where the OD at 450 nm (OD450) was 1.0, and antibody values were expressed in ELISA units (EU) which were calculated as the ratio of the OD450 generated by the test antibody to the OD450 of the standard [33,34]. A fixed IgG concentration (50 ng/ml) was used as the basis for determination of the difference in antibody levels [34]. Antibodies that bound to all tested heterologous PvDBPII (TH4, -TH5, -P, -7.18) and homologous PvDBPII-TH2 variants were considered as broadly reactive MBC-derived IgG. Anti-MSP1P-19 antibody served as a negative control, while supernatant from non-stimulated cells was used as a background control. The experiment was done in duplicate wells and repeated twice.

### Analysis of IgG subclass responses to PvDBPII-TH2 variant

To identify the IgG subclasses in the culture supernatant of specific MBC clones that are positive for anti-PvDBPII-TH2 antibodies, indirect ELISA was used to detect IgG1, IgG2, IgG3 and IgG4 antibodies specific to PvDBPII-TH2 [52]. The 96-well plates were coated with 2 μg/ml recombinant antigen. Supernatant was added at 2000 ng/mL of total IgG to duplicate wells. After washing, mouse anti-human antibodies at dilution 1/500 for IgG1 and IgG3, and 1/1000 for IgG2 and IgG4 (Invitrogen Corp) were added. Then, horseradish peroxidase (HRP)-conjugated goat anti-mouse IgG (Biolegend) at 1/500 for IgG1 and IgG3, and at 1/1,000 for IgG2 and IgG4, were used for detection. The OD of the reaction was measured at 450 nm after the addition of the tetramethylbenzidine (TMB) enzyme substrate (Sigma Aldrich). The positive controls were supernatant (n = 2) from antigen-specific MBC clones which had the highest OD value for total IgG. A baseline OD was established using supernatant from non-stimulated cells. The experiment was done in duplicate wells and repeated twice.

### Inhibition of DBPII-erythrocyte binding using COS7 assay

Expression plasmid constructs were engineered to target different variants of PvDBPII alleles (TH2, TH4, TH5, P, 7.18 and Sal I; S3 Table) on the surface of transiently transfected COS7 cells as fusion proteins to the N terminus of enhanced green fluorescent protein (EGFP). PvDBPII-erythrocyte binding inhibition assays (EBIA) were performed as previously described [14]. Briefly, duplicate wells of transfected COS7 cells were preincubated with different concentrations of clone-derived IgG from culture supernatants prior to the addition of 10% Duffy-positive human erythrocytes. Rosettes were counted in at least 30 fields of view at magnification 20x. Inhibitory function was calculated as the percentage of rosettes in the presence of tested antibody test wells relative to negative control wells. Any antibody with inhibitory activity greater than or equal to 80% was considered as highly inhibitory. 2D10 mouse monoclonal anti-PvDBPII antibody [34] and culture media from non-B cell wells were used as positive and negative controls of erythrocyte inhibition, respectively. The EBIA experiment was performed in duplicates and repeated twice. Broadly neutralizing antibodies against a panel of PvDBPII variants were identified based on the 50% inhibitory concentration (IC50) values of MBC-derived HuMoAbs. Limiting dilutions of each monoclonal antibody were tested in a range of concentrations to give 100% to 0% PvDBPII erythrocyte binding inhibition against the homologous PvDBPII-TH2 strain. Then each antibody, at its IC50, was tested for broad inhibition against a panel of PvDBPII variants by EBIA (as described above). Antibodies that inhibited binding of all tested heterologous (PvDBPII-TH4, -TH5, -P, -7.18) and homologous (PvDBPII-TH2) DBPII variants were considered as broadly neutralizing antibodies.

### Immunoglobulin gene sequencing

Immunoglobulin gene sequencing was performed as previously described [53,54]. V(D)J rearrangements of cultured specific MBCs were amplified by a nested PCR. Briefly, total RNA was extracted from frozen cell pellets of individual clones using the RNeasy Micro Kit (Qiagen) and reverse-transcribed using SuperScript III (Thermo Fisher Scientific). Synthesized cDNA was subjected to two rounds of PCR using HotStarTaq DNA Polymerase with established primers. PCR conditions (for both primary and secondary PCR) were 95°C for 15 min, followed by 43 cycles at 94°C for 30 s, 58°C (IgH) for 20 s or 60°C (IgL) for 20 s, and 72°C for 1 min. V(D)J amplified products were gel purified, and the V(D)J rearrangements were analyzed by Sanger sequencing (Azenta) and identified with IgBlast tool (https://www.ncbi.nlm.nih.gov/igblast/).

### Production of recombinant human monoclonal antibodies

Recombinant HuMoAbs were produced as described previously with slight modification [53,54]. Briefly, variable regions of the immunoglobulin heavy and light chains from single-cell cultures were cloned into human IgG1 heavy chain, heavy chain CH1, kappa, or lambda light-chain expression vectors by Gibson assembly (NEB). Pairs of heavy chain and light chain vectors were co-expressed on the Expi293 system (Thermo Fisher Scientific); IgG1 was purified with a protein G column (Thermo Fisher Scientific).

### Bio-layer interferometry (BLI)

For recombinant Fab protein production, variable regions of the immunoglobulin heavy chain were cloned into human heavy chain CH1 expression vectors. Pairs of heavy chain CH1 vectors and light chain vectors were co-expressed on the Expi293 system, and Fab protein was purified using a Talon column (Clontech). The binding affinities of the Fab proteins against PvDBPII-TH2 were measured in BLI using Octet R8 (Sartorius) and SAX biosensors (Sartorius). All reagents were prepared in 10x running buffer (Sartorius) diluted to 1x with water. The biosensors were soaked with buffer for 600 s prior to loading ligand. After biotinylated PvDBPII-TH2 were loaded at 5 μg/mL for 120 s, the sensors were washed in buffer for 60 s followed by soaking in another buffer for 120 s to measure baseline. Then, association of serially diluted Fab proteins for 300 s followed by dissociation in buffer for 600 s were measured. The responses were normalized with reference sensors and reference sample, were smoothened with Savitzky-Golay method, and were fitted to global 1:1 binding model to obtain kinetic parameters.

### Data analysis

Paired data were analyzed with Wilcoxon test. Unpaired data were analyzed with Mann-Whitney test. When multiple comparisons were done, Dunn’s multiple comparisons test was performed. Distributions of antibody and inhibition concentrations of each culture supernatant were compared among the different alleles tested for statistically significant differences by one-way ANOVA analysis of variance, and multiple-comparison analysis by Dunn’s multiple comparisons using GraphPad Prism 10 software (GraphPad). P values < 0.05 were considered statistically significant and indicated by asterisks: * p < 0.05, ** p < 0.01, *** p < 0.001, **** p < 0.0001.

## Supporting information

S1 Fig Correlation analysis between age and percentage of PvDBPII-TH2-specific atypical MBCs (aMBCs). The analysis was performed by Spearman correlation. The straight line represents the trend of correlation. Spearman r and p-value for each correlation are indicated.
(TIFF)

S2 Fig Levels of IgG subclasses in culture supernatant of PvDBPII-TH2-specific atypical MBCs (aMBC) and classical MBC clones. (A) Supernatants from anti-PvDBPII-TH2 IgG antibody-positive aMBC clones (n = 6) and (B) classical MBC clones (n = 16) were detected for IgG subclasses (IgG1, IgG2, IgG3, and IgG4) using ELISA. Bars represent average + SD. The experiment was done in duplicate wells and repeated twice.
(TIFF)

S3 Fig The inhibition of PvDBPII binding to erythrocytes by atypical and classical MBC-derived human monoclonal antibodies (HuMoAbs). HuMoAbs A1F12, A3F12, B4E06, and B4E11 specific to PvDBPII-TH2 were tested for their inhibitory activity against the homologous PvDBPII-TH2 antigen by EBIA (COS7 cells). Dashed line represented an 80% cut-off value for high inhibition.
(TIFF)

S4 Fig Heterogeneity of *P*. *vivax*-specific aMBCs. (**A**) The displayed gating strategy identified antigen-specific MBCs in *P*. *vivax* patients. PvDBPII-TH2 specific IgD^-^MBCs were classified into atypical (CD21^-^ CD27^-^) and classical (CD21^+^ CD27^+^) MBCs. CD11c and FcRL5 were used to classify the atypical MBC subpopulation. (**B**) The expression of CD11c and FcRL5 on specific aMBCs. (-) Indicates negative expression (MFI: < 1000 for CD11c, < 500 for FcRL5, (+) indicates low expression (MFI: 1000–2000 for CD11c, 500–1000 for FcRL5), (++) indicates medium expression (MFI: 2000–4000 for CD11c, 1000–2000 for FcRL5), (+++) indicates high expression (MFI: > 4000 for CD11c, > 2000 for FcRL5).
(TIFF)

S5 Fig Purified recombinant PvDBPII variant antigens examined by SDS-PAGE analysis. SDS-PAGE gel of PvDBPII-TH2, -TH4, -TH5, -P, -7.18 and reference strain Sal I.
(TIFF)

S1 Table Kinetic parameters for binding affinity of HuMoAbs to PvDBPII-TH2 variant. (DOCX)

S2 Table Percentage of CDR3 similarity of atypical MBC (aMBC) and classical MBC clones. (DOCX)

S3 Table Demographic information of enrolled subjects. (DOCX)

S4 Table Panel of PvDBPII alleles used for protein expression and COS7 EBIA assay. Polymorphic residues within PvDBPII and positions with reference to Sal I (Bold) are indicated. Conserved residues are represented by a dot (.).
(DOCX)

S5 Table Marker panel used for flow cytometry. (DOCX)

S1 File Raw data for Figs 1–6 and S1-S4 of this study. (XLSX)
