# Supplementary figures and images for: Atypical memory B cells from natural malaria infection produced broadly neutralizing antibodies against Plasmodium vivax variants
Source: PLoS Pathog. 2025 Jan 23;21(1):e1012866. doi: 10.1371/journal.ppat.1012866 (PMC11756785; doi:10.1371/journal.ppat.1012866)

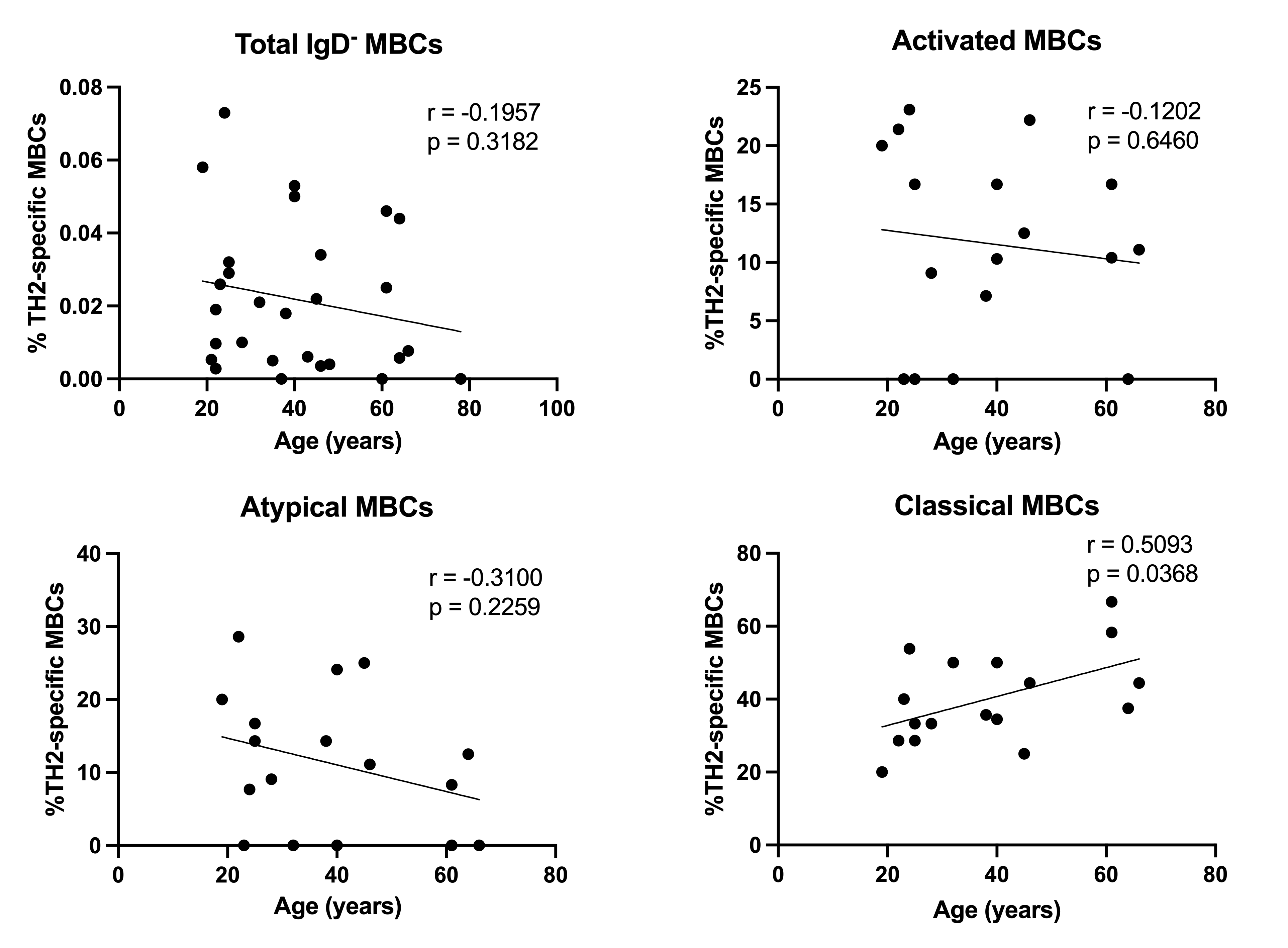

Supplement: S1 Fig — The analysis was performed by Spearman correlation. The straight line represents the trend of correlation. Spearman r and p-value for each correlation are indicated. (TIFF) [file ppat.1012866.s001.tiff]

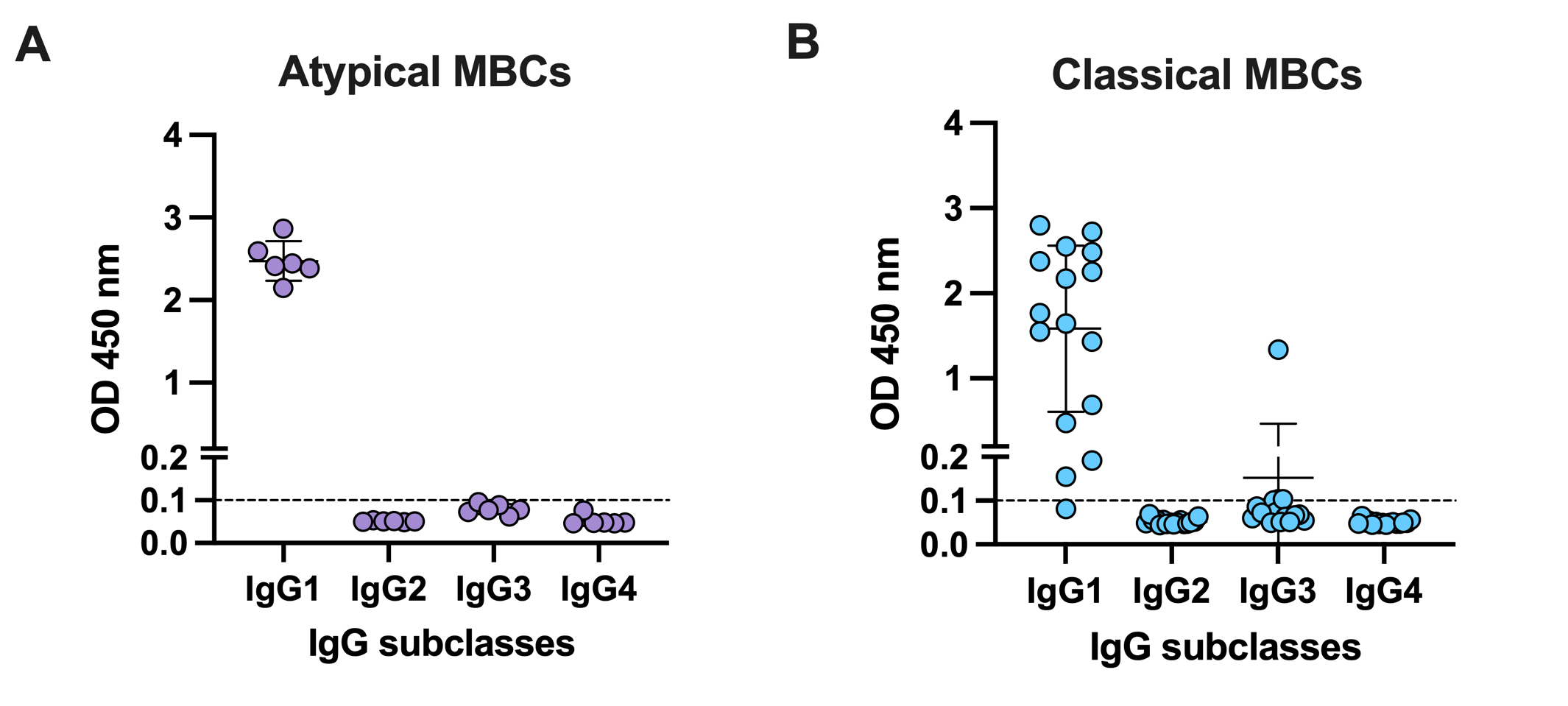

Supplement: S2 Fig — (A) Supernatants from anti-PvDBPII-TH2 IgG antibody-positive aMBC clones (n = 6) and (B) classical MBC clones (n = 16) were detected for IgG subclasses (IgG1, IgG2, IgG3, and IgG4) using ELISA. Bars represent average + SD. The experiment was done in duplicate wells and repeated twice. (TIFF) [file ppat.1012866.s002.tiff]

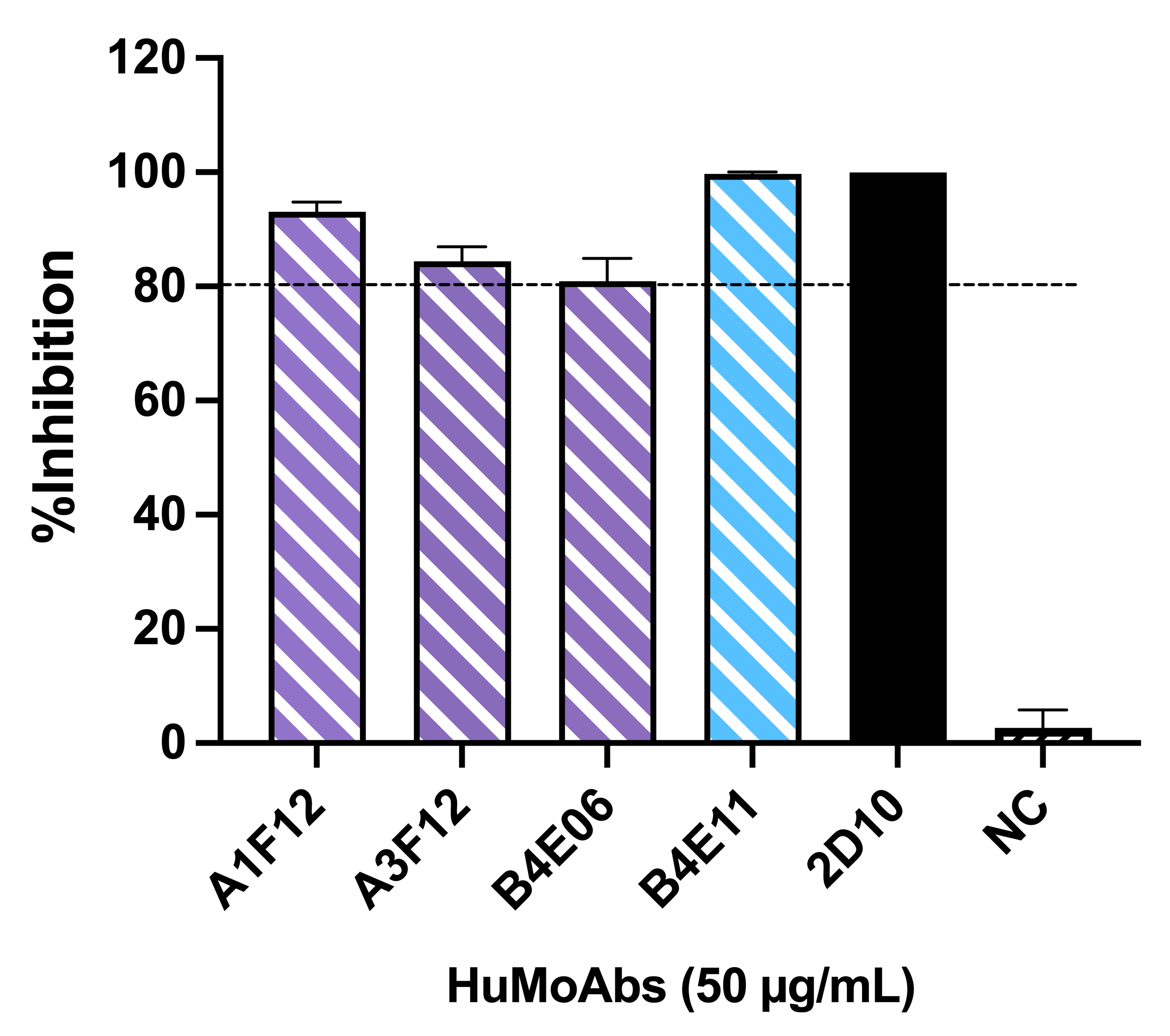

Supplement: S3 Fig — HuMoAbs A1F12, A3F12, B4E06, and B4E11 specific to PvDBPII-TH2 were tested for their inhibitory activity against the homologous PvDBPII-TH2 antigen by EBIA (COS7 cells). Dashed line represented an 80% cut-off value for high inhibition. (TIFF) [file ppat.1012866.s003.tiff]

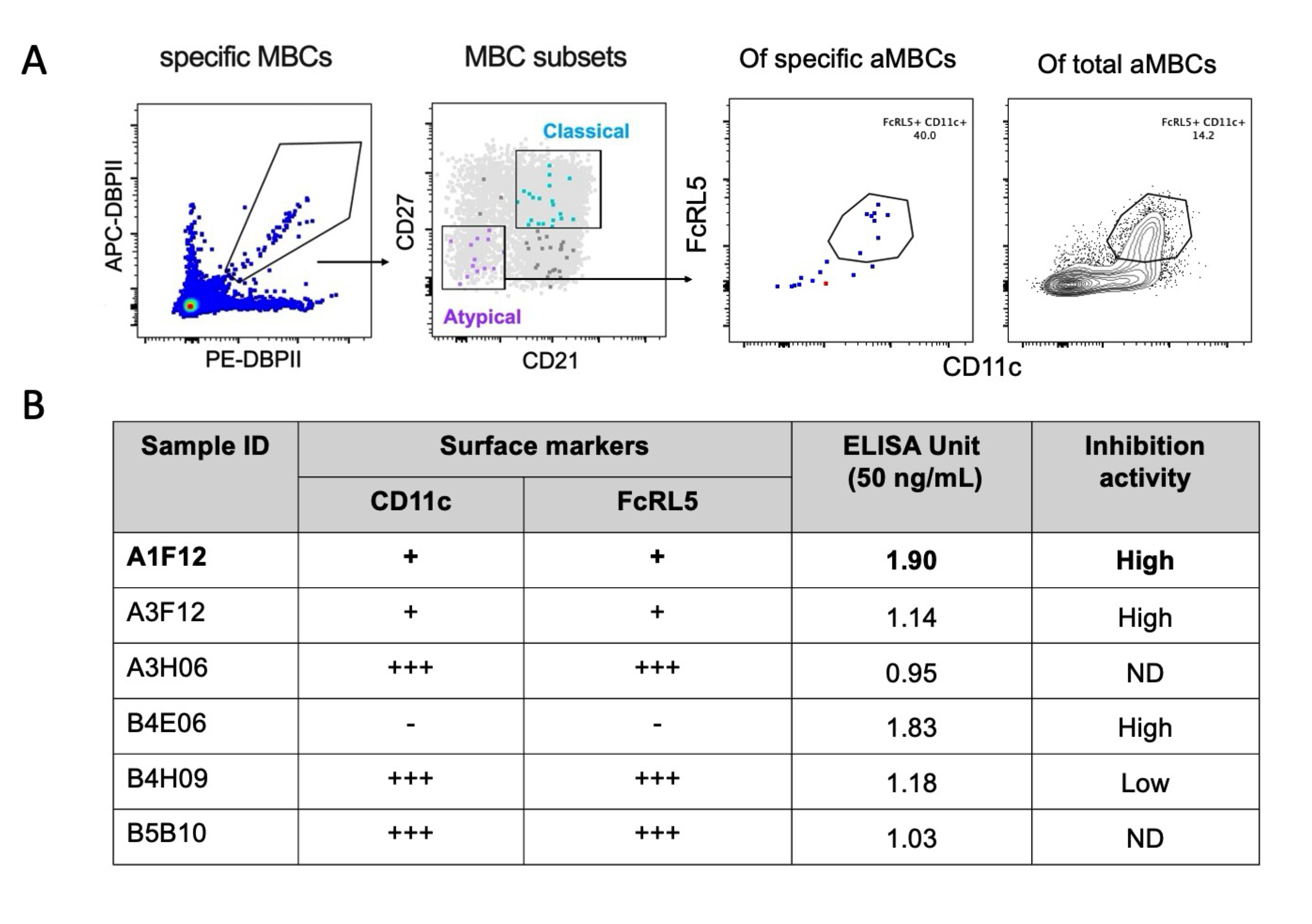

Supplement: S4 Fig — (A) The displayed gating strategy identified antigen-specific MBCs in P. vivax patients. PvDBPII-TH2 specific IgD-MBCs were classified into atypical (CD21- CD27-) and classical (CD21+ CD27+) MBCs. CD11c and FcRL5 were used to classify the atypical MBC subpopulation. (B) The expression of CD11c and FcRL5 on specific aMBCs. (-) Indicates negative expression (MFI: < 1000 for CD11c, < 500 for FcRL5, (+) indicates low expression (MFI: 1000–2000 for CD11c, 500–1000 for FcRL5), (++) indicates medium expression (MFI: 2000–4000 for CD11c, 1000–2000 for FcRL5), (+++) indicates high expression (MFI: > 4000 for CD11c, > 2000 for FcRL5). (TIFF) [file ppat.1012866.s004.tiff]

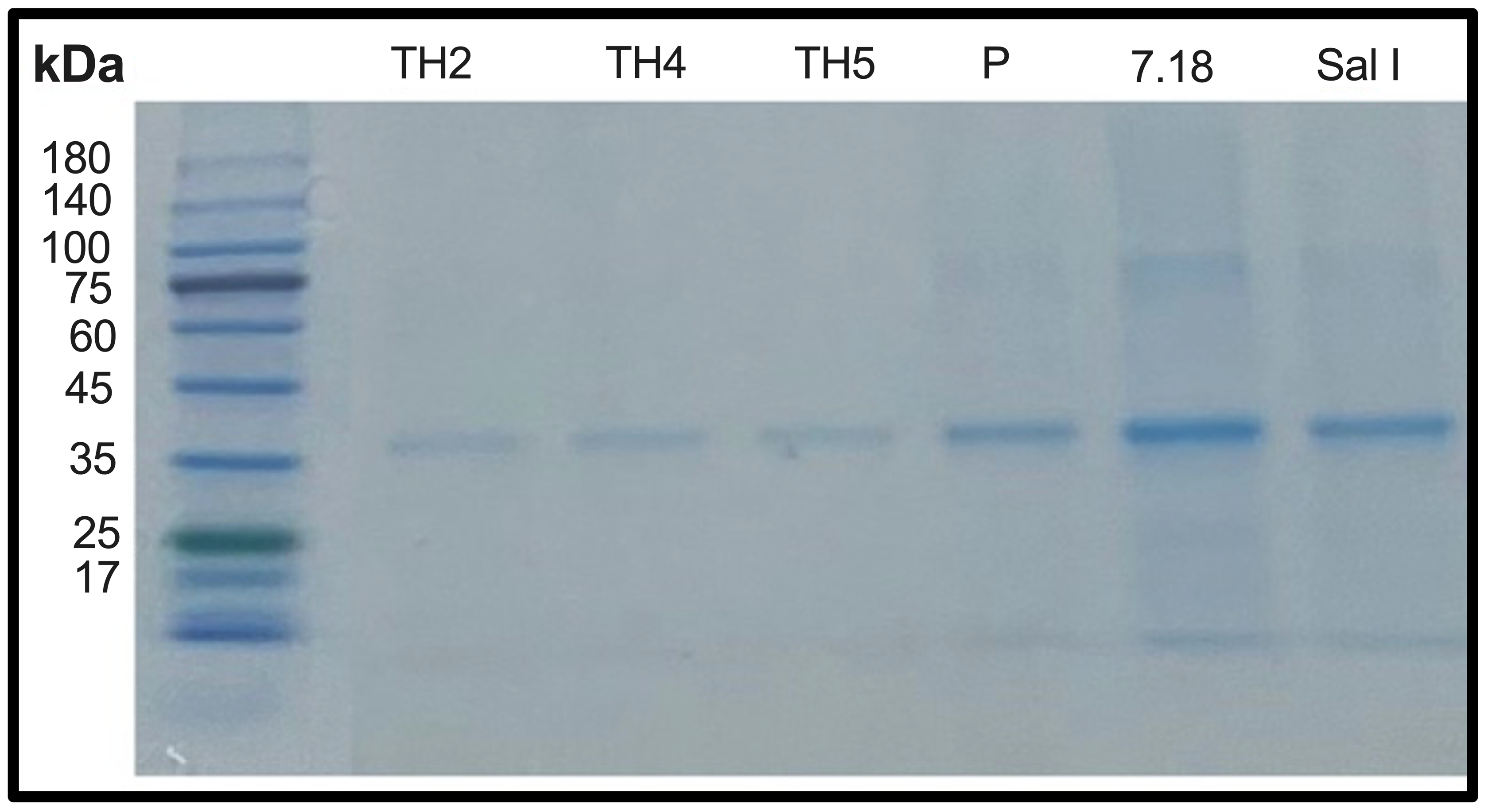

Supplement: S5 Fig — SDS-PAGE gel of PvDBPII-TH2, -TH4, -TH5, -P, -7.18 and reference strain Sal I. (TIFF) [file ppat.1012866.s005.tiff]
