# Supplementary material for: Atypical memory B cells from natural malaria infection produced broadly neutralizing antibodies against Plasmodium vivax variants
Source: PLoS Pathog. 2025 Jan 23;21(1):e1012866. doi: 10.1371/journal.ppat.1012866 (PMC11756785; doi:10.1371/journal.ppat.1012866)
Supplement: S1 Table — (DOCX) [file ppat.1012866.s006.docx]

**S1 Table. Kinetic parameters for binding affinity of HuMoAbs to PvDBPII-TH2 variant.**

| **Origin** | **Sample ID** | ***K_D_* (M)** | ***k_on_* (1/Ms)** | ***k_off_* (1/s)** |
| --- | --- | --- | --- | --- |
| **Atypical** | A1F12 | 4.6 ± 4.3 ×10^-11^ | 2.1 ± 0.2 ×10^5^ | 1.0 ± 1.0 ×10^-5^ |
| **Atypical** | A3F12 | 2.6 ± 1.3 ×10^-8^ | 5.5 ± 0.4 ×10^5^ | 1.4 ± 0.8 ×10^-2^ |
| **Atypical** | B4E06 | 4.7 ± 0.7 ×10^-9^ | 5.3 ± 0.5 ×10^5^ | 2.5 ± 0.5 ×10^-4^ |
| **Classical** | B4E11 | < 1 ×10^-12^ | 4.0 ± 0.5 ×10^5^ | < 1 ×10^-7^ |
