# Supplementary material for: Atypical memory B cells from natural malaria infection produced broadly neutralizing antibodies against Plasmodium vivax variants
Source: PLoS Pathog. 2025 Jan 23;21(1):e1012866. doi: 10.1371/journal.ppat.1012866 (PMC11756785; doi:10.1371/journal.ppat.1012866)
Supplement: S2 Table — (DOCX) [file ppat.1012866.s007.docx]

**S2 Table. Percentage of CDR3 similarity of atypical MBC (aMBC) and classical MBC clones.**

| **IgGHV** | **IgG clones** | **% CDR3 similarity** |
| --- | --- | --- |
| **IGHV1-3** | A3F12 and A3H06 | 87.5% |
|  | A3F12 and A2B04 | 100% |
|  | A3H06 and A2B04 | 87.5% |
| **IGHV1-69** | B4H09 and B5B10 | 38.46% |
|  | B4H09 and B4G04 | 23.52% |
|  | B5B10 and B4G04 | 30.77% |
