# Supplementary material for: Atypical memory B cells from natural malaria infection produced broadly neutralizing antibodies against Plasmodium vivax variants
Source: PLoS Pathog. 2025 Jan 23;21(1):e1012866. doi: 10.1371/journal.ppat.1012866 (PMC11756785; doi:10.1371/journal.ppat.1012866)
Supplement: S3 Table — (DOCX) [file ppat.1012866.s008.docx]

**S3 Table. Demographic information of recruited subjects.**

| **Characteristics** | ***P. vivax* specific MBC analysis** | | **Healthy subjects** |
| --- | --- | --- | --- |
|  | **Acute *P. vivax* patients** | **Recovery subjects (6-9 months)** |  |
| Total (n) | 28 | 15 | 27 |
| Parasitemia (parasite/μL)  Mean ± SD (range) | 3451.9 ± 1958.5  (990.5– 5742.6) | 0 | 0 |
| Ages  Median (IQR) | 39 (26.25) | 39.00 (27.50) | 28.00 (7.50) |
| Gender | | | |
| Male | 19 | 7 | 13 |
| Female | 9 | 8 | 14 |
| Nationality | | | |
| Thai | 24 | 15 | 27 |
| Myanmar | 4 | 0 | 0 |
| No. of prior infection | | | |
| 0 | 27 | 14 | - |
| 1 | 1 | 1 | - |
| No. of recorded re-infection | 1 | 1 | - |
| Time intervals for reinfection | 12 months | 3 months | - |
