# Supplementary material for: Atypical memory B cells from natural malaria infection produced broadly neutralizing antibodies against Plasmodium vivax variants
Source: PLoS Pathog. 2025 Jan 23;21(1):e1012866. doi: 10.1371/journal.ppat.1012866 (PMC11756785; doi:10.1371/journal.ppat.1012866)
Supplement: S4 Table — Polymorphic residues within PvDBPII and positions with reference to Sal I (Bold) are indicated. Conserved residues are represented by a dot (.). (DOCX) [file ppat.1012866.s009.docx]

**S4 Table. Panel of DBPII alleles used for protein expression and COS7 EBIA assay.** Polymorphic residues within PvDBPII and positions with reference to Sal I (Bold) are indicated. Conserved residues are represented by a dot (.).

| **DBPII allele** | **Residue position** | | | | | | | | | | | | | | |
| --- | --- | --- | --- | --- | --- | --- | --- | --- | --- | --- | --- | --- | --- | --- | --- |
|  | **308** | **313** | **333** | **371** | **375** | **384** | **385** | **386** | **390** | **417** | **424** | **433** | **437** | **475** | **503** |
| **DBPII Sal I** | **R** | **.** | **L** | **K** | **.** | **D** | **E** | **K** | **R** | **N** | **L** | **.** | **W** | **P** | **I** |
| DBL-TH2 | . | . | F | E | . | G | K | Q | . | K | I | . | R | . | . |
| DBL-TH4 | . | . | F | . | . | G | K | Q | H | K | I | . | R | . | K |
| DBL-TH5 | . | . | . | E | . | G | . | N | . | K | I | . | R | . | K |
| DBPII-P | S | . | F | . | . | G | K | N | H | K | I | . | R | . | K |
| DBPII-7.18 | S | . | . | . | . | G | . | Q | . | K | I | . | R | . | K |
