# Supplementary material for: Atypical memory B cells from natural malaria infection produced broadly neutralizing antibodies against Plasmodium vivax variants
Source: PLoS Pathog. 2025 Jan 23;21(1):e1012866. doi: 10.1371/journal.ppat.1012866 (PMC11756785; doi:10.1371/journal.ppat.1012866)
Supplement: S5 Table — (DOCX) [file ppat.1012866.s010.docx]

**S5 Table. Marker panel used for flow cytometry.**

|  | Marker | Fluorochrome | Clone | Company |
| --- | --- | --- | --- | --- |
| 1 | CD19 | FITC | HIB19 | BioLegend |
| 2 | CD19 | BUV395 | HIB19 | BD Biosciences |
| 3 | CD21 | PeCPcy5.5 | Bu32 | BioLegend |
| 4 | CD21 | BV786 | Bu32 | BD Biosciences |
| 5 | CD27 | APC/fire | M-T271 | BioLegend |
| 6 | CD27 | BV605 | M-T271 | BD Biosciences |
| 7 | IgD | PE/Cy7 | IA6-2 | BioLegend |
| 8 | IgD | BUV737 | IA6-2 | BD Biosciences |
| 9 | IgG | BV421 | G18-145 | BD Biosciences |
| 10 | CD2 | BV510 | RPA-2.10 | BD Biosciences |
| 11 | CD3 | AF-700 | OKT3 | BioLegend |
| 12 | CD4 | BV510 | RPA-T4 | BD Biosciences |
| 13 | CD10 | BV510 | HI10a | BD Biosciences |
| 14 | CD14 | AF-700 | M5E2 | BioLegend |
| 15 | CD14 | BV510 | M5E2 | BD Biosciences |
| 16 | CD16 | AF-700 | 3G8 | BioLegend |
| 17 | FCRL5 | BV650 | 509F6 | BD Biosciences |
| 18 | CD11c | BUV615 | 3.9 | BD Biosciences |
| 19 | PvDBL-TH2 | PE | - | Invitrogen |
| 20 | PvDBL-TH2 | APC | - | Invitrogen |
| 21 | Live/dead aqua | - | - | Invitrogen |
| 22 | Zombie RedTM Fixable Viability Kit | - | - | BioLegend |
